# Supplementary material for: Informal caregiver training for people with chronic pain in musculoskeletal services (JOINT SUPPORT): protocol for a feasibility randomised controlled trial
Source: BMJ Open. 2023 Jan 27;13(1):e070865. doi: 10.1136/bmjopen-2022-070865 (PMC9884939; doi:10.1136/bmjopen-2022-070865)
Supplement: Author's manuscript [file bmjopen-2022-070865.draft_revisions.pdf]

BMJ Open

BMJ Open is committed to open peer review. As part of this commitment we make the peer review history of every article we publish publicly available.

When an article is published we post the peer reviewers' comments and the authors' responses online. We also post the versions of the paper that were used during peer review. These are the versions that the peer review comments apply to.

The versions of the paper that follow are the versions that were submitted during the peer review process. They are not the versions of record or the final published versions. They should not be cited or distributed as the published version of this manuscript.

BMJ Open is an open access journal and the full, final, typeset and author-corrected version of record of the manuscript is available on our site with no access controls, subscription charges or pay-per-view fees (http://bmjopen.bmj.com).

If you have any questions on BMJ Open's open peer review process please email info.bmjopen@bmj.com

BMJ Open

Informal caregiver training for people with chronic musculoskeletal pain (JOINT SUPPORT): protocol for a feasibility randomised controlled trial

Journal:	*BMJ Open*
Manuscript ID	bmjopen-2022-070865
Article Type:	Protocol
Date Submitted by the Author:	06-Dec-2022
Complete List of Authors:	Smith, Toby; University of Warwick; University of East Anglia<br>Khoury, Reema; University of East Anglia, Faculty of Medicine and Health Sciences<br>Ashford, Polly-Anna; University of East Anglia, Faculty of Medicine and Health Sciences<br>Hanson, Sarah; University of East Anglia, Faculty of Medicine and Health Sciences<br>Welsh, Allie; University of East Anglia, School of Education<br>Clark, Allan; University of East Anglia, Faculty of Medicine and Health Sciences<br>Dures, Emma; University of the West of England, School of Health and Social Wellbeing; Bristol Royal Infirmary, Academic Rheumatology<br>Adams, Jo; University of Southampton, School of Health Sciences; University of East Anglia, Faculty of Medicine and Health Sciences
Keywords:	PAIN MANAGEMENT, REHABILITATION MEDICINE, RHEUMATOLOGY

SCHOLARONE™
Manuscripts

BMJ OPEN

TITLE PAGE

Title:

Informal caregiver training for people with chronic musculoskeletal pain (JOINT SUPPORT): protocol for a feasibility randomised controlled trial

Authors:

Smith TO,[1,2] Khoury R,[2] Ashford P,[2] Hanson S,[2] Welsh A,[3] Clark A,[2] Dures E,[4,5] Adams J[2,6]

Affiliations:

1. Warwick Medical School, University of Warwick, Coventry, UK
2. Faculty of Medicine and Health Sciences, University of East Anglia, Norwich, UK
3. School of Education, University of East Anglia, Norwich, UK
4. Academic Rheumatology, Bristol Royal Infirmary, Bristol, UK
5. School of Health and Social Wellbeing, University of the West of England, Bristol, UK
6. School of Health Sciences, University of Southampton, Southampton, UK

Corresponding Author:

Professor Toby Smith, Warwick Clinical Trials Unit, Warwick Medical School, University of Warwick, Coventry, CV4 7AL, UK. Email: toby.o.smith@warwick.ac.uk

Word Count: Abstract: 298; Manuscript: 4341

For peer review only

ABSTRACT

Introduction: Chronic musculoskeletal (bone, joint or muscle) pain is disabling. People with it frequently have difficulties in managing everyday activities. Individuals may rely on family members or friends to support them. These people are known as informal caregivers. No interventions have previously addressed the health needs of people with chronic musculoskeletal pain and their caregivers. In response, the JOINT SUPPORT programme was developed. In this study, we will assess the feasibility and acceptability of conducting a pragmatic, multi-centre randomised controlled trial (RCT) to test the clinical and cost-effectiveness of the JOINT SUPPORT programme to support these individuals.

Methods and Analysis: This will be a mixed-methods feasibility RCT. We will recruit 80 patients with chronic musculoskeletal pain with their informal caregivers. Patients will be randomised to usual National Health Service (NHS) care *OR* usual NHS care plus a caregiver-patient dyad training programme (JOINT SUPPORT). This programme comprises of five, one-hour, group-based sessions for patients and caregivers, delivered by trained physiotherapists or occupational therapists. It includes developing skills in: understanding pain, pacing, graded activity, fear avoidance and goal-setting, understanding benefits of physical activity and skills in medication management. This will be re-enforced with a workbook. After the group-based sessions, patients and caregivers will be supported through three telephone sessions with a therapist. Data collected at baseline and three months will include: screening logs, intervention logs, fidelity checklists and clinical outcomes on quality of life, physical and emotional outcomes, adverse events and resource use. Qualitative research with 24 patient-caregiver dyads and 12 health professionals will explore the acceptability of trial processes. Stop-go criteria will inform the progression to a full trial.

Ethics and Dissemination: Ethical approval was obtained on 22th February 2022 (National Research Ethics Committee Number: 22/NW/0015). Results will be reported at conferences, peer-review publications and across social media channels.

Trial Registration Number: ISRCTN registration: ISRCTN78169443

Status: Trial recruitment is on-going and the study is expected to be completed by June 2024.

Keywords: Pain; Caregiver: Dyad; RCT; Process Evaluation; Rehabilitation

STRENGTHS AND LIMITATIONS OF THIS STUDY

1. JOINT SUPPORT is a pragmatic, multicentre, feasibility RCT.
2. This study will determine if it is feasible to conduct an RCT to assess the effectiveness of a caregiving intervention for people with chronic musculoskeletal pain.
3. The sample size is sufficient to inform decision-making on the design of a larger full-trial.
4. The embedded qualitative study will explore experiences and views on of the study design and intervention.
5. The relative small sample size increases the risk of higher potential loss to follow-up.

For peer review only

INTRODUCTION

Chronic musculoskeletal (bone, joint or muscle) pain is disabling. It is seen in all ages of people[1]. It encompasses conditions such as low back pain, neck pain, fibromyalgia, osteoarthritis, pain from fractures or other rheumatological diseases. Many people have more than one body region affected[2]. It affects approximately 17 million people in the UK, with 9.1 million people living in England with long-term back pain[3]. NHS costs to treat musculoskeletal diseases are in excess of £5 billion per year[3].

People with chronic musculoskeletal pain frequently have difficulties in managing their symptoms and everyday activities to maintain independence and quality of life[4]. To assist with symptoms, they often access support. This may include help in tasks such as: washing and dressing, preparing meals and assistance in feeding, housework or shopping[5,6]. This caregiving may be formal or informal. Formal care is defined as the provision of care by someone who is paid. Informal care is provided without a direct payment. This is often given by a spouse or partner, family members and/or friends[7].

Being a caregiver for someone with chronic musculoskeletal pain can be a physical and emotional burden[8]. Becoming a caregiver in this situation can significantly change the dynamic of a relationship with feelings of burden which can led to resentment[9], changing roles and identities not only between the patient-caregiver dyad but also impacts on family, social and occupational life, and fear and concern regarding the future and how chronic pain may impact on the individual they support, over-time[9,10]. Usual NHS care is focused on the patient, providing interventions to support the long-term management of pain and disability. These are either through structured programmes such as the ESCAPE-Pain programme[11], or non-structured guidelines incorporating elements of education, exercise, pain relief and psychological interventions[12-14]. In both instances, none of these approaches has included caregiver interventions to support symptom management.

No caregiving interventions have existed to address the health and social needs of people with chronic musculoskeletal pain or to support caregivers. In response, the research team developed the JOINT SUPPORT programme. This is aimed to improve patient symptom management by educating, supporting and empowering caregivers to optimise the support they give their family member or friend with chronic musculoskeletal pain.

Caregiver interventions, in principle, improve the health and wellbeing of patients and their caregivers through better self-management skills[9]. This is important as living with pain is associated with a negative health status, including reduced independence, social isolation and loneliness, obesity and comorbidities associated with physical inactivity such as type two diabetes, depression and cardiovascular disease[15-17]. Improving the skills, capability, motivation, confidence and knowledge so that people with chronic musculoskeletal pain can better manage their symptoms could reduce demand on NHS services through improved self-management and wider health improvement.

AIM AND OBJECTIVES

AIM: To assess the feasibility of conducting a pragmatic, multi-centre randomised controlled trial (RCT) to test the clinical and cost-effectiveness of an informal caregiver training programme to support people with chronic musculoskeletal pain.

OBJECTIVES:

The main objectives are to assess the:

For peer review only - http://bmjopen.bmj.com/site/about/guidelines.xhtml

- Feasibility of recruiting eligible patient and caregiver dyads in the NHS.
- Acceptability of the JOINT SUPPORT programme to caregivers, patients and health professionals.
- Fidelity of delivery of the JOINT SUPPORT programme by health professionals.
- Ability of caregivers to deliver components of the JOINT SUPPORT programme with fidelity, confidence and competency to patients at home.
- Acceptability of caregiving dyad randomisation for patients, caregivers and health professionals.
- Risk of intervention contamination when experimental and control groups are delivered in the same setting.

The secondary objectives are to assess the:

- Completeness of outcome measure data.
- Signal of the clinical effectiveness of the JOINT SUPPORT programme.

METHODS AND ANALYSIS

Trial Design

A mixed-methods feasibility study comprising of a parallel, multicentre, pragmatic RCT and embedded qualitative study. **Figure 1** illustrates the study flow.

Prior to commencing, all participating site team members will be taught the skills and knowledge required to fulfil their delegated role in the trial. They will have completed training, including Good Clinical Practice (GCP)[18] if required. Their participation and competence in delegated roles will be recorded in a site delegation log.

Eligibility

We will include:

- Patients aged 18 years and over with a history (six weeks or more) of pain from a musculoskeletal (bone, joint or muscle) origin who have a current referral to, or who are attending physiotherapy, rheumatology, orthopaedic, occupational therapy or pain management services.
- Patients able to nominate an informal caregiver. An informal caregiver is defined as someone who has done or is expected to provide unpaid care, assistance, support or supervision in activities of daily living for at least three hours per week over two or more personal contacts.
- Patients and caregivers willing and able to provide consent.
- Patients and caregivers who can engage in a group-based intervention currently delivered in English.

For peer review only

If the participating hospital appointment is routinely a virtual appointment rather than in-person, patient and caregiver participants must have access to a device which can receive this If a patient has multiple caregivers, a single, nominated by the patient, 'principal' caregiver will be elected.

We will exclude:

- Patients or caregivers with acute (requiring hospitalisation) or terminal illness (life expectancy < six weeks) which would make participation in the rehabilitation strategies contraindicated and/or impractical.

Recruitment

Patients will be identified and approached during rheumatology, orthopaedic, pain management, occupational therapy or physiotherapy out-patient appointments. A member of the clinical team will screen participants against the eligibility criteria. This will be recorded on the site screening log. For eligible participants, a member of the site clinical team will briefly outline the study and offer a Recruitment Information Pack. The pack includes: covering letter, Participant Information Sheet (PIS, separate for patient and caregiver), Consent Form (patient and caregiver), Baseline Questionnaires (separate for patient and caregiver), Participant Contact Details Form (separate for patient and caregiver) and Demographic Characteristics Form (patient and caregiver) with a pre-paid envelope. With this information, potential participants will be asked to consider participation. If a caregiver is present with the patient at the appointment, they will be provided with the caregiver PIS. If not, the patient will be provided with this to give to the caregiver. The potential patient and caregiver participant will be asked to read this information. After a minimum of 24 hours, each participant provided with the Recruitment Information Pack will be telephoned or video-called by the site team member and provided with an opportunity to ask questions. They will be informed that if they would like to participate, they should complete the Consent Form, and then (sequentially) Participant Contact Details Form (patient and caregiver) and Demographic Characteristics Form (patient and caregiver) and Baseline Questionnaire (patient and caregiver) and to return them using the pre-paid envelope to the Central Trial Team, who will then randomise the participant-dyad and notify the site team of both enrolment and group allocation. We will aim to recruit potential patient and caregiver participants within 14 days of the initial contact.

All screening activity will be recorded by a member of the trained local site team using the trial's screening log. Eligible participants who are approached but who do not wish to participate will be anonymously recorded as part of a screening log, providing information on: age, gender, date of appointment and type of musculoskeletal pathology.

Both caregiver and patient consent will be required.

Randomisation, blinding and allocation concealment

Randomisation will take place once patient and caregiver participant consent forms and baseline questionnaire packs have been completed, received and checked by a member of the Central Trial Team at Norwich Clinical Trials Unit (NCTU). The randomisation scheme will be generated by the NCTU Trial Manager and allocated group will be notified by email to the site trial team. Randomisation will be stratified at the individual dyad level (approximately 2:1) by:

- Site

For peer review only

- Age of patient (< or > and equal to 65 years).

The allocation is computer generated so will not be known prior to randomisation. Allocation is concealed prior to randomisation to prevent selection (or treatment allocation) bias.

Due to the participatory nature of rehabilitation, it will not be possible to blind patients, caregivers or treating health professionals to group allocation after randomisation. The trial PIS has presented intervention equipoise. Therefore, whilst not possible to blind, we have aimed to limit expectation bias through careful selection of PIS wording for participants.

Intervention

Usual Care

This will be National Health Service (NHS) treatment as usual (TAU). Participant dyads randomised to this group will receive standard NHS care delivered by their department e.g., physiotherapy, rheumatology, orthopaedic, occupational therapy or pain management services. This consists of patient-focused treatments which may include exercise, medication prescription and advice/education[11-14]. This may be face-to-face or through video consultation approaches depending on local NHS service provision. There is no routine 'training' element or hands-on skills or formal support for caregivers. Patients and their caregivers will not receive the JOINT SUPPORT programme or any caregiver training.

Treatment logs will be used to record the components of standard care. This will be completed by the clinical team to determine what interventions are received by the patient whilst they are under the care of that service.

Experimental Intervention

The JOINT SUPPORT programme was developed from the evidence-base[9] and following patient, caregiver and stakeholder input. The programme is developed and informed by Social Cognitive Theory[19]. This is an explanation for behaviour in which individual learning happens in a social context, where people can put vicarious learning into practice, and learn together and from each other in the process. The three goals of the intervention are outlined below using a CONTEXT-MECHANISM-OUTCOME framework (**Figure 2**)[20] and logic model (**Figure 3**).

To improve caregiver knowledge, capability and skills by identifying and practising individualised chronic pain management strategies and physical activity participation in out-patient (or video consultation) settings that can be used for people with MSK pain

Caregivers of people with chronic musculoskeletal pain (CONTEXT) need the skills, capability opportunity and knowledge (MECHANISM) to be able to support and guide behaviour change for both patients and caregivers to increase health-related quality of life (HRQoL), reduce social isolation and improve self-management outcomes for patients (OUTCOME).

To reduce caregiver fear of movement and isolation when providing care for people with chronic pain

Chronic musculoskeletal pain leads to fear of movement, isolation and a loss of identity for caregivers (CONTEXT) requiring a re-evaluation of their role and identity (MECHANISM) to be able to support

For peer review only

people with chronic musculoskeletal pain and promote good caregiver health and wellbeing (OUTCOME).

Support patients and caregivers to increase capability, set targets and monitor goals to facilitate patients improved symptom management

Patients with chronic musculoskeletal pain in an out-patient setting (CONTEXT) will be supported by caregivers to set personalised shared goals which they can realistically meet (MECHANISM) to facilitate improved functional, health-related outcomes and increased independence (OUTCOME).

Participants randomised to the JOINT SUPPORT programme will receive usual NHS treatment (control group intervention as detailed below) PLUS five, one-hour, group-based training sessions, delivered by a study-trained physiotherapist or occupational therapist. The intervention will be delivered through two possible approaches.

Approach 1: Face-to-face delivery which will be group-based, consisting of a target three to five dyads in each class. They will be delivered in an out-patient setting provided to both participants in the dyad by a study-trained physiotherapist or occupational therapist.

Approach 2: Video consultation if face-to-face consultations are not permitted within a participating NHS organisation, group-based video consultation sessions using an NHS authorised platform will be used to deliver the content of the JOINT SUPPORT programme as per the face-to-face delivery approach.

If there is a change in service delivery once a participant-dyad has started their JOINT SUPPORT programme, switching from Approach 1 to Approach 2 will be permitted, with the effects explored in the qualitative sub-study.

JOINT SUPPORT Training Programme

The first session will aim to start within six weeks from consent. Sessions will be provided to both patient and caregiver participants by either a physiotherapist or occupational therapist. Irrespective of delivery method, each session will be up to 60 minutes. Throughout the five sessions, the JOINT SUPPORT health professional will monitor the patient-caregiver competencies, providing continual feedback and critique to support the training. There will be a cognitive-behavioural approach used throughout the sessions, underpinned by social cognitive theory where dyads will be facilitated by a JOINT SUPPORT health professional on the following topics:

Session 1:

- Understanding pain, caregiving and how pain affects the caregiving dyad
- Introduction and explanation of the JOINT SUPPORT Workbook, highlighting material on pain and effects on the dyad

Session 2:

- Pacing and graded activity
- Goal setting
- JOINT SUPPORT Workbook - highlighting material on goal-setting and problem-solving

Session 3:

For peer review only

- Benefits of physical activity (reducing deconditioning, healthy ageing, physical and psychological health)
- Fear avoidance
- JOINT SUPPORT Workbook - highlighting material on physical activity and fear avoidance

Session 4:

- Medication use and management
- JOINT SUPPORT Workbook - highlighting material on medication use and management

Session 5:

- Working through case-study scenarios to re-enforce knowledge and critique competencies on JOINT SUPPORT skills
- JOINT SUPPORT Workbook - highlighting material on case-scenarios and long-term goal setting
- Confirmation of dates for JOINT SUPPORT Telephone Booster calls

JOINT SUPPORT Telephone Booster Calls

Following the five group-sessions, a JOINT SUPPORT health professional will telephone each caregiver and patient as a dyad during Week 1, 3 and 6 post-group session discharge. Each call is expected to take approximately 20 minutes. Both the caregiver and patient participant should be in the same room during these calls. Topics covered in each call will include:

- Recovery progress and current status based on patient-caregiver shared goals
- Discussion on JOINT SUPPORT Workbook use and progress including goal-setting sheets
- Support to create collaborative goals and positive reinforcement for continued recovery

JOINT SUPPORT Healthcare Professional Training

Designated health professionals delivering the experimental intervention will attend a one-day face-to-face course where, at the local participating site, they will be taught the JOINT SUPPORT intervention by a member of the research team. In addition, to assess the fidelity to the experimental intervention, the trial team will undertake a debriefing telephone/video call with health professional after they have delivered their first JOINT SUPPORT session.

Contamination

Participants will only receive their allocated intervention. During follow-up, participants may require further interventions as part of their recovery as per routine NHS practice. Further clinical interventions will be permitted for participants without having to withdraw from the trial, but these will be recorded by the Central Trial Team. To assess for the risk of crossover between groups, we will closely monitor case report forms (CRFs) and data pertaining to intervention delivery.

Co-interventions

This is a pragmatic study and patient-caregiver dyads in either group will not be asked to desist from receiving other forms of treatment during the study such as continuing rehabilitation, general practitioner (GP) consultations, medication changes or alternative treatments as required. Use of these treatments will be recorded through CRFs.

Quality Assessment

For peer review only

The trial will be monitored and audited in accordance with the current approved protocol, principles of GCP[18], relevant regulations and Standard Operating Procedures (SOPs).

A quality control programme will be adopted to ensure protocol and intervention fidelity. We will collect data on what components of the interventions (control and experimental) were delivered. This is in respect of intervention parameters including: content, mode of delivery, staff delivered, frequency, timing of delivery and variation/deviations from the protocol. These will be collected through intervention logs and relevant CRFs. Quality Assurance (QA) checks through site visits will be conducted at Month 1 from first randomisation (+/- three weeks for each) at each site. These visits may be in-person or virtual. If there are concerns in relation to any aspect of the site visit, repeat visits with training may be undertaken to improve protocol compliance.

Assessment

The data collection schedule is summarised in **Figure 4**.

To answer our feasibility objectives, we will assess:

- Recruitment feasibility – by screening log data: number of potential patients and caregivers screened, assessed for eligibility; including reasons for exclusion/non-participation, and consented.
- Intervention acceptability – by conducting qualitative interviews with participants; recording study attrition at the intervention phase; analysing acceptability questionnaires for patients, caregivers and health professionals.
- Intervention fidelity (health professionals) – by analysis and reporting on intervention log checklist data on: intervention timing, duration, frequency, timing of intervention (during or after 'active' course of treatment); QA monitoring visit checklists; qualitative interviews.
- Intervention fidelity (caregivers) – by analysis and reporting on caregiver intervention logs; qualitative interviews.
- Randomisation acceptability – by analysis and reporting on screening logs, eligibility assessment logs and consent forms; participant attrition; qualitative interviews.
- Contamination risk - by analysis and reporting on intervention log data including: experimental and control intervention records; QA monitoring visit checklists; delegation logs; qualitative interviews (patient, caregiver and healthcare professional).

Secondary outcomes

- Outcome data completeness – by analysis and reporting on completion rates (baseline and three months post- randomisation) of: *Patient Participants*: Musculoskeletal-Health Questionnaire (MSK-HQ)[21]; numerical rating scale for pain and fatigue[22] ; self-efficacy, assessed using the General Self-Efficacy Scale (GSE)[23]; psychological distress (depression), assessed using the Center for Epidemiologic Studies Depression Scale (CES-D)[24]; HRQoL assessed using the EQ-5D-5L[25]; self-reported health resource use questionnaire; adverse events. *Caregiver Participants*: EQ-5D-5L[25]; CES-D [24]; the Zarit Burden Interview Scale – 12-item version[26] Leisure Time Satisfaction questionnaire (LTS)[27]; self-reported health resource use questionnaire; adverse events.

- Signal of effectiveness – by calculating and reporting effect size measurement of the above outcome measures.

These measures were selected due to their robust psychometric properties and acceptable patient and caregiver burden as confirmed by our study Patient Representatives/clinician feedback. They reflect relevant core outcome sets including: lower limb osteoarthritis[28], low back pain[29] and general musculoskeletal disease[30]. Caregiver outcomes mirror the domains of importance reported through previous qualitative findings[9].

Baseline assessment will be undertaken after patient and caregiver participants have signed the consent form, prior to randomisation. Patient and caregiver participants will be provided with a paper-based questionnaire to complete in their Recruitment Information Pack. Baseline data collected will include: (for patient and caregiver): age, sex, ethnicity, occupational status (current or past if retired), medical co-morbidities, presenting musculoskeletal pathology(ies); and for patient, duration of symptoms relationship of caregiver to patient; and for caregiver: duration of caregiving, whether caregiver for another person, whether lives with patient.

At three months post-randomisation, patient and caregiver participants will be sent a postal questionnaire. A three-month follow-up period will provide an indication on follow-up completion and usability of the outcome instruments to answer a trial secondary outcome measure. Participants will be asked to complete and return this to the Central Trial Team using a pre-paid envelope. If participants do not return these questionnaires within two weeks of initial posting, the Central Trial Team will telephone the participants (caregiver and/or care-recipient) to offer the option of completing the questionnaires over the telephone or to send a further questionnaire pack.

Data Analysis

Sample Size

In total, 80 participant dyads (80 patients/80 caregivers) will be recruited. This sample size will be sufficient to answer our feasibility objectives and assess the *a priori* progression criteria (**Table 1**)[31]. This number will also allow each site to test the JOINT SUPPORT programme in at least two complete cycles. This sample size follows recommendations[32] for designing feasibility trials that aim to detect a small-medium standardised effect size (where the definitive trial will be designed with 80% power and two-sided 5% significance)[31,33].

Analysis

Consent rates, recruitment rates, attrition, missing data rates and intervention fidelity will be reported as proportions with 95% confidence intervals (CIs). The analysis of clinical outcome measures will be descriptive, reported as means and standard deviations or medians and interquartile ranges if not normally distributed for continuous outcomes and numbers and percentages for binary and categorical variables. Between-group mean differences will be reported together with 95% CIs. No formal statistical testing will be undertaken.

Health Economic

Data on health care utilisation will be collected but not analysed. To answer the feasibility questions related to the health economic perspectives, we will test the completion of the health resource use questionnaire and will present the data descriptively.

For peer review only - http://bmjopen.bmj.com/site/about/guidelines.xhtml

Progression Criteria

A 'traffic light' system (**Table 1**) will be used as a guide for progression to a definitive trial[34]. If any criteria are not met, they will be discussed by the Trial Oversight Committee (TOC) to decide if a definitive trial is feasible with, or without, refinements.

Qualitative study

The embedded qualitative investigation will assess intervention and study design acceptability for patients, caregivers and health professionals.

Eligibility

The embedded qualitative study is optional for participants. Patient and caregivers will be able to express an interest to be approached regarding the qualitative sub-study on the main study consent form at the beginning of the study. The sample will consist of patient-caregiver dyads and physiotherapists and occupational therapists who have delivered the JOINT SUPPORT intervention.

Dyads who have agreed to be contacted for an interview will be purposively sampled to ensure a maximum variation in: age, gender, presenting musculoskeletal pathology, ethnicity, duration of disability, severity of disability (as measured by the baseline MSK-HQ[21], relationship of caregiver to patient and duration the caregiver has been caring for the patient. Our intention is for caregivers and patients to be interviewed separately to enable them to more freely express their views but we will interview as a dyad if this is expressly requested.

We anticipate around twenty-four interviews will be conducted, involving approximately 12 patients/12 caregivers from the JOINT SUPPORT programme and approximately eight caregivers/eight patients from the TAU group across the sites. Based on our previous research[9], this sample size should ensure a range of different viewpoints to reach information power[35] to answer our feasibility questions.

The health professionals delivering the JOINT SUPPORT intervention will be invited to participate in the voluntary interviews within three months of completing one participant through the JOINT SUPPORT programme. A minimum target of one physiotherapist and one occupational therapist who delivered the intervention will be invited to be interviewed from each site (eight participants in total). This will provide a range of contexts from different professional backgrounds. The health professionals will be sent a PIS outlining the objectives and processes involved with this optional sub-study. They will be asked to contact the research team by email if they are interested in participating. Those who express an interest will be contacted to arrange a mutually convenient time to conduct the interview.

Data Collection

Interviews will aim to be conducted up to six weeks post-intervention via telephone or video call, at a time convenient to dyads and healthcare professionals. This allows exploration and reflection of the patients' and caregivers' study experience once the intervention has ended in a reasonable recall period. Interviews will be semi-structured, following an open-ended question schedule, with a maximum duration of 60 minutes. Open-ended questions will explore the acceptability of the research based on Sekhon et al's[36] acceptability framework including the values of the intervention, burden,

For peer review only

perceived effectiveness, self-efficacy and suggestions for improvement from their perspectives. Interviews will be conducted by a qualitative researcher (AW), closely supervised by a senior qualitative researcher (SH).

Prior to conducting the interview, consent will be obtained and audio recorded from the participant. The interview will then follow a semi-structured, open-ended question schedule (**Table 2; Table 3**). Broadly, participants will be asked their attitudes towards topics including the:

- Timing, frequency, duration and content of the JOINT SUPPORT training programme and telephone booster calls
- Support and training provided for the group-based programme and telephone boosters
- Recording of both components of the JOINT SUPPORT programme
- Logistic of managing the participant flow
- Their views on the recruitment process
- Acceptability of the JOINT SUPPORT programme
- Potential modifications or recommended changes to the JOINT SUPPORT programme
- Perceived effectiveness/helpfulness

Data Analysis

All interviews will be audio-recorded, transcribed and anonymised. After transcription, the audio data will be destroyed. Data will be analysed thematically taking a two-stage approach to understand the important contextual factors that have influenced the implementation of JOINT SUPPORT. We aim to initially analyse all data deductively, guided by the MRC guidance for complex interventions[37-39], to assess the quality of implementation, clarify the hypothesised causal mechanisms identified in our logic model (for example, goal setting in the training and the support provided by the telephone coaching), identify contextual factors associated with variation in outcomes and how the intervention might be optimised for acceptability[36]. Data will then be analysed more inductively and more broadly. This will include critiquing the conceptual approach of JOINT SUPPORT, understanding any unintended consequences and reflections on the JOINT SUPPORT intervention from the health professional, patient and caregiver perspective.

Trial Status

The trial is funded for 24 months commencing in April 2022. Recruitment is expected to be complete by June 2023 with the final follow-up visit completed by November 2023. The trial will be completed by April 2024.

Patient and Public Involvement

Patients have been involved with the study development from inception. Their involvement will continue throughout the trial. A patient-dyad member will attend TOC meetings. They will provide insights into the trial conduct, particularly on data collection processes. They will also help interpret the findings and the dissemination phase.

For peer review only

ETHICS AND DISSEMINATION

All data will be processed according to the Data Protection Act[40] (2018). All documents will be stored safely in confidential conditions. Trial-specific documents, except for the signed consent form and follow-up contact details, will refer to the participant with a unique study participant number, not by name. Participant identifiable data will be stored separately from trial data. All trial data will be stored securely in offices or online in secure trial databases, only accessible by the Central Trial Team and authorised personnel. Participant withdrawal is permitted at any point post-enrolment. This will be recorded on a trial CRF, including reasons for withdrawal. Adverse events and Serious Adverse Events will be recorded and reported in-accordance to ICH GCP[18].

The results of the trial will be reported using the CONSORT guidelines[41] including the relevant extensions for non-pharmacological interventions and for pilot and feasibility studies. The intervention will be reported according to the TIDIER guidelines[42]. This will be presented to academic, clinical and patient and public audiences. To ensure that outputs are accessible to diverse stakeholder groups, we will develop result materials across our research team including patient representatives.

For peer review only

ACKNOWLKEDGEMENTS AND DECLARATIONS

JOINT SUPPORT Collaborators: Mrs Coralie Crowther (Norfolk, UK), Mrs Shelia Macaulay-Cripps (Norwich, Norfolk), Mr John Macaulay-Cripps (Norwich, Norfolk), Jessica Pawson, Joanna Fletcher (Principle Investigator) and Catherine Hilton (Barts Health NHS Trust, London), Gregory Booth (Royal National Orthopaedic Hospital NHS, Trust, London - Principle Investigator), Gareth Stephens (The Royal Orthopaedic Hospital NHS Foundation Trust, Birmingham – Principle Investigator), Caroline Swain (The Royal Orthopaedic Hospital NHS Foundation Trust, Birmingham), Sarah Rich (The Royal Orthopaedic Hospital NHS Foundation Trust, Birmingham) and Chris Drake (Principle Investigator) and Tom Shelton (Mid-Yorkshire Hospitals NHS Trust, Wakefield).

Trial Oversight Committee Members: Professor Patricia Schofield (Chair) (University of Plymouth), Professor Lindsay Bearne (St George's University, London), Dr Catherine Best (University of Stirling). Trial Oversight Committee Charter can be accessed through reasonable request to the Corresponding Author.

Trial Sponsor: University of East Anglia, Norwich, NR4 7TJ, UK (Email: rin.fmh@uea.ac.uk).

Funding: This study is funded by the National Institute for Health Research (NIHR) Research for Patient Benefit grant (NIHR202723). The views expressed are those of the authors and not necessarily those of the NIHR or the Department of Health and Social Care.

*Neither the funder or sponsor have ultimate authority over the analysis, interpretation, writing of reports or submission of publications.

Protocol Number: Protocol Version 3.1 – Dated 15 September 2022

Contributions: TOS, RK, PAA, SH, AW, AC, ED, JA developed the study concept and design; TOS, ED, JA provide clinical expertise; SH, AW led the qualitative research design; AC led the statistical analysis design; TOS, AC, RK, PAA, SH, ED, JA provided trial design and conduct expertise; TOS, RK, PAA, SH, AW, AC, ED, JA drafted the protocol paper and agreed the submission; TOS acts as guarantor.

Ethical Approvals: Ethical approval was obtained on 22nd February 2022 (National Research Ethics Committee Number: 22/NW/0015).

Trial Registration Number: ISRCTN registration: ISRCTN78169443

Competing Interests: None declared

Patient consent for publication: Not required.

Provenance and Peer-Review: Not commissioned: externally peer-reviewed

Data availability statement: Data will be available on reasonable request. Access to the de-identified dataset for purposes of research other than this study, will be at the discretion of the Chief Investigator, Professor Toby Smith and NCTU. Requests for the de-identified dataset generated during the current study should be made to the Chief Investigator, Professor Toby Smith (email: toby.o.smith@warwick.ac.uk) or NCTU (email: hiphelper@uea.ac.uk). Professor Toby Smith and NCTU will consider requests once the main results from the study have been published up until 31 June 2027.

For peer review only

FIGURE AND TABLE LEGENDS

Figure 1: Study flow-chart illustrating the participant flow for the JOINT SUPPORT study

Figure 2: Figure illustrating the Context-Mechanism-Outcome framework for the JOINT SUPPORT intervention.

Figure 3: Figure illustrating the JOINT SUPPORT intervention logic model.

Figure 4: Participant timeline illustrating schedule of enrolment, interventions, and assessments.

Table 1: Feasibility study stop-go, traffic light, progression criteria

Table 2: Interview topic guide for the caregiving dyad interviews

Table 3: Interview topic guide for the Healthcare Professional Interviews

For peer review only

REFERENCES

1. Smith E, Hoy DG, Cross M, *et al*. The global burden of other musculoskeletal disorders: estimates from the Global Burden of Disease 2010 study. *Ann Rheum Dis* 2014;**73**:1462-9.

2. Raja R, Dube B, Hensor EM, *et al*. The clinical characteristics of older people with chronic multiple-site joint pains and their utilisation of therapeutic interventions: data from a prospective cohort study. *BMC Musculoskelet Disord* 2016;**17**:194.

3. Public Health England. Health Profile for England. Public Health England 2021. Available at: https://fingertips.phe.org.uk/static-reports/health-profile-for-england/hpfe_report.html Accessed on: 06Dec2022

4. Wolff JL, Spillman BC, Freedman VA, Kasper JD. A national profile of family and unpaid caregivers who assist older adults with health care activities. *JAMA Intern Med* 2016;**176**:372-9.

5. Whybrow P, Moffatt S, Kay L, Thompson B, Aspray T, Duncan R. Assessing the need for arthritis training among paid carers in UK residential care homes: A focus group and interview study. *Musculoskeletal Care* 2018;**16**:82-9.

6. Riffin C, Van Ness PH, Wolff JL, Fried T. Family and other unpaid caregivers and older adults with and without dementia and disability. *J Am Geriatr Soc* 2017;**65**:1821-8.

7. Smith TO, Mansfield M, Hanson S, *et al*. Caregiving for older people living with chronic pain: analysis of the English Longitudinal Study of Ageing and Health Survey for England. *Br J Pain* 2022; In Press.

8. Darragh AR, Sommerich CM, Lavender SA, *et al*. Musculoskeletal discomfort, physical demand, and caregiving activities in informal caregivers. *J Appl Gerontol* 2015;**34**:734–60.

9. Smith T, Fletcher J, Lister S. Lived experiences of informal caregivers of people with chronic musculoskeletal pain: a systematic review and meta-ethnography. *Br J Pain* 2021;**15**:187-98.

10. Richardson JC, Ong BN, Sim J. Experiencing chronic widespread pain in a family context: giving and receiving practical and emotional support. *Sociol Health Illn* 2007;**29**:347–65.

11. Hurley MV, Walsh NE, Mitchell HL, *et al*. Clinical effectiveness of a rehabilitation program integrating exercise, self-management, and active coping strategies for chronic knee pain: a cluster randomized trial. *Arthritis Rheum* 2007;**57**:1211-9.

12. NICE. Osteoarthritis: care and management - 2014. Guideline CG 177. Accessed: 06Dec2022. Available at: https://www.nice.org.uk/guidance/cg177

13. NICE. Chronic pain (primary and secondary) in over 16s: assessment of all chronic pain and management of chronic primary pain - 2021. Guideline NG193. Accessed: 06Dec2022. Available at: https://www.nice.org.uk/guidance/ng193

14. NICE. Low back pain and sciatic in over 16s: assessment and management – 2020. Guideline NG59. Accessed: 06Dec2022. Available at: https://www.nice.org.uk/guidance/ng59

15. Katz J, Rosenbloom BN, Fashler S. Chronic pain, psychopathology, and DSM-5 somatic symptom disorder. *Can J Psychiatry* 2015;**60**:160-7.

16. Hassoon A, Bydon M, Kerezoudis P, Maloney PR, Rinaldo L, Yeh HC. Chronic low-back pain in adult with diabetes: NHANES 2009-2010. *J Diabetes Complications* 2017;**31**:38-42.

17. Fayaz A, Ayis S, Panesar SS, Langford RM, Donaldson LJ. Assessing the relationship between chronic pain and cardiovascular disease: A systematic review and meta-analysis. *Scand J Pain* 2016;**13**:76-90.

18. ICH. 2022. ICH Harmonisation for Better Health. Accessed: 06Dec2022. Available at: https://www.ich.org/

19. Bandura A. *Social foundations of thought and action: A social cognitive theory*. Prentice-Hall, Inc, 1986.

20. Pawson R, Tilley N. *Realistic evaluation*. London: SAGE; 1997.

21. Hill JC, Kang S, Benedetto E, *et al*. Development and initial cohort validation of the Arthritis Research UK Musculoskeletal Health Questionnaire (MSK-HQ) for use across musculoskeletal care pathways. *BMJ Open* 2016;**6**:e012331.

22. Farrar JT, Young JP Jr, LaMoreaux L, Werth JL, Poole MR. Clinical importance of changes in chronic pain intensity measured on an 11-point numerical pain rating scale. *Pain* 2001;**94**:149-58.

23. Schwarzer R, Jerusalem M. Generalized Self-Efficacy scale. In J. Weinman, S. Wright, & M. Johnston, *Measures in health psychology: A user's portfolio. Causal and control beliefs* (pp. 35-37). Windsor, UK: NFER-NELSON, 1995.

24. Radloff LS. The CES-D scale: A self-report depression scale for research in the general population. *Appl Psycholog Measurement* 1977;**1**:385-401.

25. EuroQol: EQ-5D. Accessed: 06Dec2022. Available at: http://www.euroqol.org/

26. Hebert R, Bravo G, Preville M. Reliability, validity and reference values of the Zarit Burden Interview for assessing informal caregivers of community-dwelling older persons with dementia. *Canadian J Aging* 2000;**19**:494-507.

27. Stevens AB, Coon D, Wisniewski S, *et al*. Measurement of leisure time satisfaction in family caregivers. *Aging Ment Health* 2004;**8**:450-9.

28. Smith TO, Hawker GA, Hunter DJ, *et al*. The OMERACT-OARSI Core Domain Set for Measurement in Clinical Trials of Hip and/or Knee Osteoarthritis. *J Rheumatol* 2019;**46**:981-9.

29. Chiarotto A, Deyo RA, Terwee CB, *et al*. Core outcome domains for clinical trials in non-specific low back pain. *Eur Spine J* 2015;**24**:1127-42.

30. Klokkerud M, Dagfinrud H, Uhlig T, et al. Developing and testing a consensus-based core set of outcome measures for rehabilitation in musculoskeletal diseases. *Scand J Rheumatol* 2018;**47**:225-34.

For peer review only

31. Teare MD, Dimairo M, Shephard N, Hayman A, Whitehead A, Walters SJ. Sample size requirements to estimate key design parameters from external pilot randomised controlled trials: a simulation study. *Trials* 2014;**15**:264.

32. Whitehead AL, Julious SA, Cooper CL, Campbell MJ. Estimating the sample size for a pilot randomised trial to minimise the overall trial sample size for the external pilot and main trial for a continuous outcome variable. *Stat Methods Med Res* 2016;**25**:1057-73.

33. Sim J, Lewis M. The size of a pilot study for a clinical trial should be calculated in relation to considerations of precision and efficiency. *J Clin Epidemiol* 2012;**65**:301–8.

34. Avery KN, Williamson PR, Gamble C, *et al*. Informing efficient randomised controlled trials: exploration of challenges in developing progression criteria for internal pilot studies. *BMJ Open* 2017;**7**:e013537.

35. Malterud K, Siersma VD, Guassora AD. Sample size in qualitative interview studies: guided by information power. *Qual Health Res* 2016;**26**:1753-60.

36. Sekhon M, Cartwright M, Francis JJ. Acceptability of healthcare interventions: an overview of reviews and development of a theoretical framework. *BMC Health Serv Res* 2017;**17**:88.

37. Craig P, Dieppe P, Macintyre S, *et al*. Developing and evaluating complex interventions: the new Medical Research Council guidance. *BMJ* 2008;**337**:a1655.

38. Moore GF, Audrey S, Barker M, *et al*. Process evaluation of complex interventions: Medical Research Council guidance. *BMJ* 2015;**350**:h1258.

39. Skivington K, Matthews L, Simpson SA, *et al*. A new framework for developing and evaluating complex interventions: update of Medical Research Council guidance. *BMJ* 2021;374:n2061.

40. Data Protection Act. 2018. Data accessed: 06Dec2022. Available at: http://www.legislation.gov.uk/ukpga/2018/12/contents/enacted.

41. Schulz KF, Altman DG, Moher D; CONSORT Group. CONSORT 2010 statement: updated guidelines for reporting parallel group randomised trials. *PLoS Med* 2010;**7**:e1000251.

42. Hoffmann TC, Glasziou PP, Boutron I, et al. Better reporting of interventions: template for intervention description and replication (TIDieR) checklist and guide. *BMJ* 2014;**348**:g1687.

Table 1: Feasibility study stop-go, traffic light, progression criteria

	Green (Go)	Amber (Amend)	Red (Stop)
**Recruitment**	>30% of patients screened across the sites in 12 months would be eligible	20% to 30% would be eligible	<20% would be eligible
**Intervention Fidelity (Health Professionals)**	>70% of participant-dyads compliant with their allocated intervention (5 face-to-face sessions and booster phone call) as randomised	50% to 70% received intervention as randomised	<50% received intervention as randomised
**Intervention Fidelity (Caregivers)**	>90% of participants adopted elements of JOINT SUPPORT programme post-last session	60% to 90% adopted JOINT SUPPORT post-last session	<60% adopted elements post-last session
**Randomisation Acceptability**	>40% of eligible participants consent to be randomised	20%-40% would be randomised	<20% would be randomised
**Contamination**	<5% of participants in either group received majority of their allocated treatment cross-over	5%-10% of participants cross-over	>10% of participants cross-over
**Data Collection Completion**	<15% missing outcome questionnaires for whatever reason at 3-month data collection	15%-30% missing questionnaires	>30% missing questionnaires

For peer review only

Table 2: Interview topic guide for the caregiving dyad interviews

The interview will be structured on the following areas of interest	Sample questions
Introduction	Overall, could you share your experiences of being involved with our research?
Determining participant views of their intervention	First of all, can you talk me through what study treatment you received? (prompt – clarify what was JOINT SUPPORT and what was usual care/non-study intervention)
The approach and consent process and willingness to be randomised to either group	Can you talk me through how you got into the study? You were allocated to *X* group. What did that feel like? Could we have dealt with that differently?
The acceptability of the care (both groups)	Would you be happy to talk me through your treatment?<br>As *X*'s carer, what was your impression of the care. For both of you, what was helpful and less helpful to your care?
Group-based JOINT SUPPORT programme and telephone booster calls (experimental group)	How far did you find the JOINT SUPPORT programme helpful – for both of you. Can you give specific examples? What didn't work as well?<br>Did you get the telephone calls from the trial team? Can you remember what you talked about? Can you give specific examples of what was helpful, and l helpful? Was there any advice that confused you or you weren't clear about?
What the strengths of the experimental intervention (perceived effectiveness)	What were the most helpful/good-bits of your JOINT SUPPORT intervention? What was good about it. What didn't you like about it?
What the weaknesses of the experimental intervention	What were the less helpful/worse bits of the JOINT SUPPORT intervention?
What modifications they may recommend to interventions received (standard care and experimental groups)	What could we improve? (prompt: What do you think is lacking?)<br>How do you think we could better support you and your carer to support you with chronic pain?
The risk of intervention contamination between the groups	Did you talk to any other patients or caregivers whilst in hospital about the intervention? Was there any discussion between those who received it and did not receive it?
The ease and convenience of the data collection processes (baseline and three months) (all participants)	As you were part of a trial, we had to collect a lot of measurements. Can you talk me through what these were? How easy were they? How convenient were they? Overall, do you have any points to make about the testing?
Applicability of the methods and measures used	How did you manage with the questionnaires we gave you at the start of the study and at the end in the post? Were they easy to complete or do you remember them being a problem?

For peer review only

Table 3: Interview topic guide for the Healthcare Professional Interviews

**The interview will be structured on the following areas of interest**	**Sample questions**
Introduction (Perceived effectiveness)	Overall, could you share your experiences of being involved with our research?
The randomised to either group	How did you feel about 50% of the patients not receiving the JOINT SUPPORT intervention but getting normal care? Did this 'sit easy' with you?
The acceptability of the group-based care	How did the delivery of the JOINT SUPPORT group sessions go? How did you work out who would do what? Was there a decision on professional background? Did you feel comfortable teaching all the content? Were any modifications made? How did the patients and caregivers get on with it in your opinion?
JOINT SUPPORT Telephone Calls	How did you feel about doing the telephone calls? Were they helpful for caregivers and patients? Was it feasible to deliver one call to both members of the dyad? Did you make any modifications to the content of the call?
Training on Intervention	Did you feel adequately prepared to deliver the group-based and telephone JOINT SUPPORT interventions? Would you recommend any changes to this? Did you need any additional 'top up' or 'refresher' training sessions?
The risk of intervention contamination between the groups	Do you think you used the JOINT SUPPORT intervention on control or non-trial patients? Did other professionals not in the trial use the intervention? If either occurred, do you think anything could have been done to avoid this?
The ease and convenience of the data collection processes	As you were part of a trial, we had to collect a lot of measurements. How easy were the intervention data collection logs? How convenient were they? What changes would you recommend if any were needed?

For peer review only

Figure 1: Study flow-chart illustrating the participant flow for the JOINT SUPPORT study

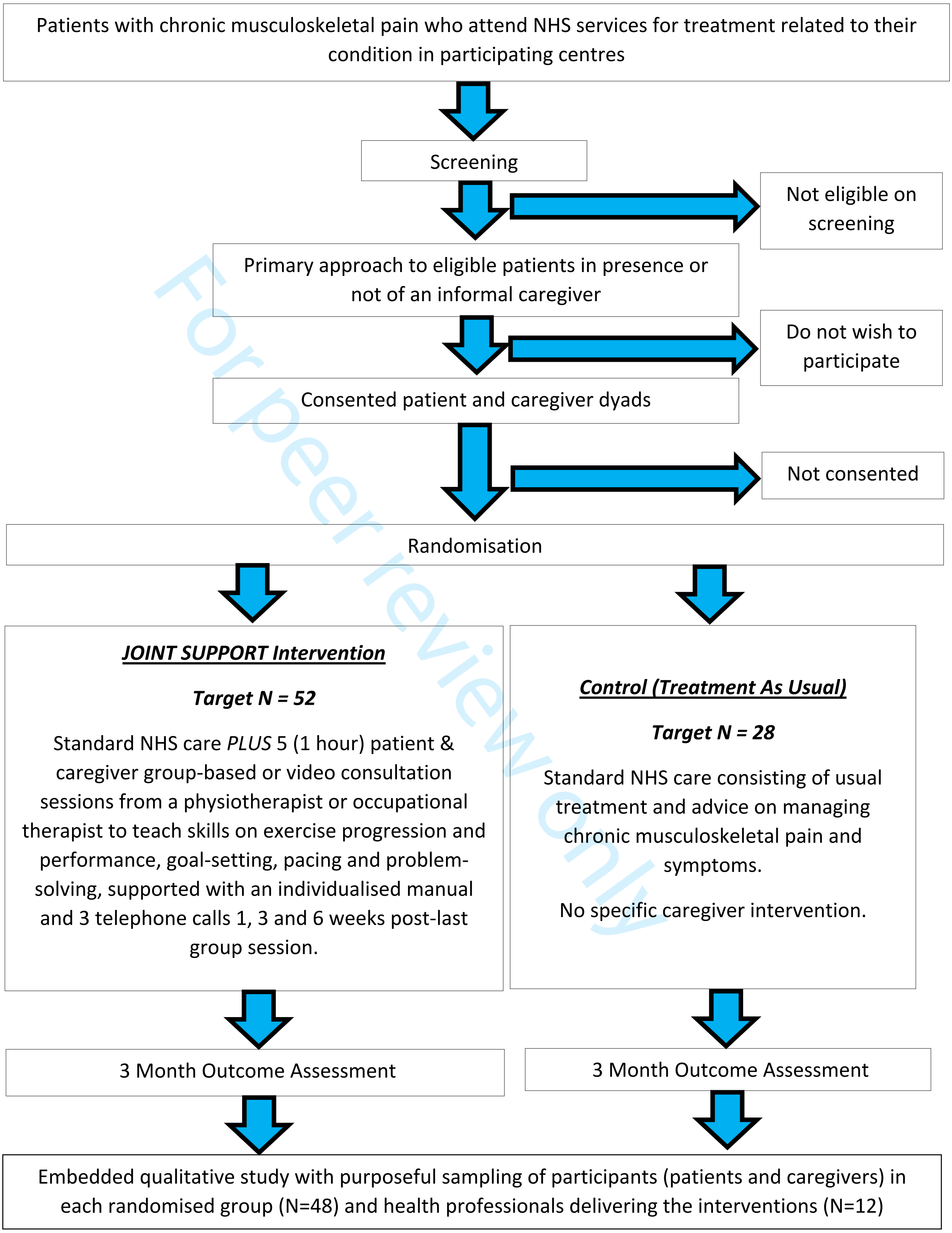

For peer review only

Figure 2: Figure illustrating the Context-Mechanism-Outcome framework for the JOINT SUPPORT intervention.

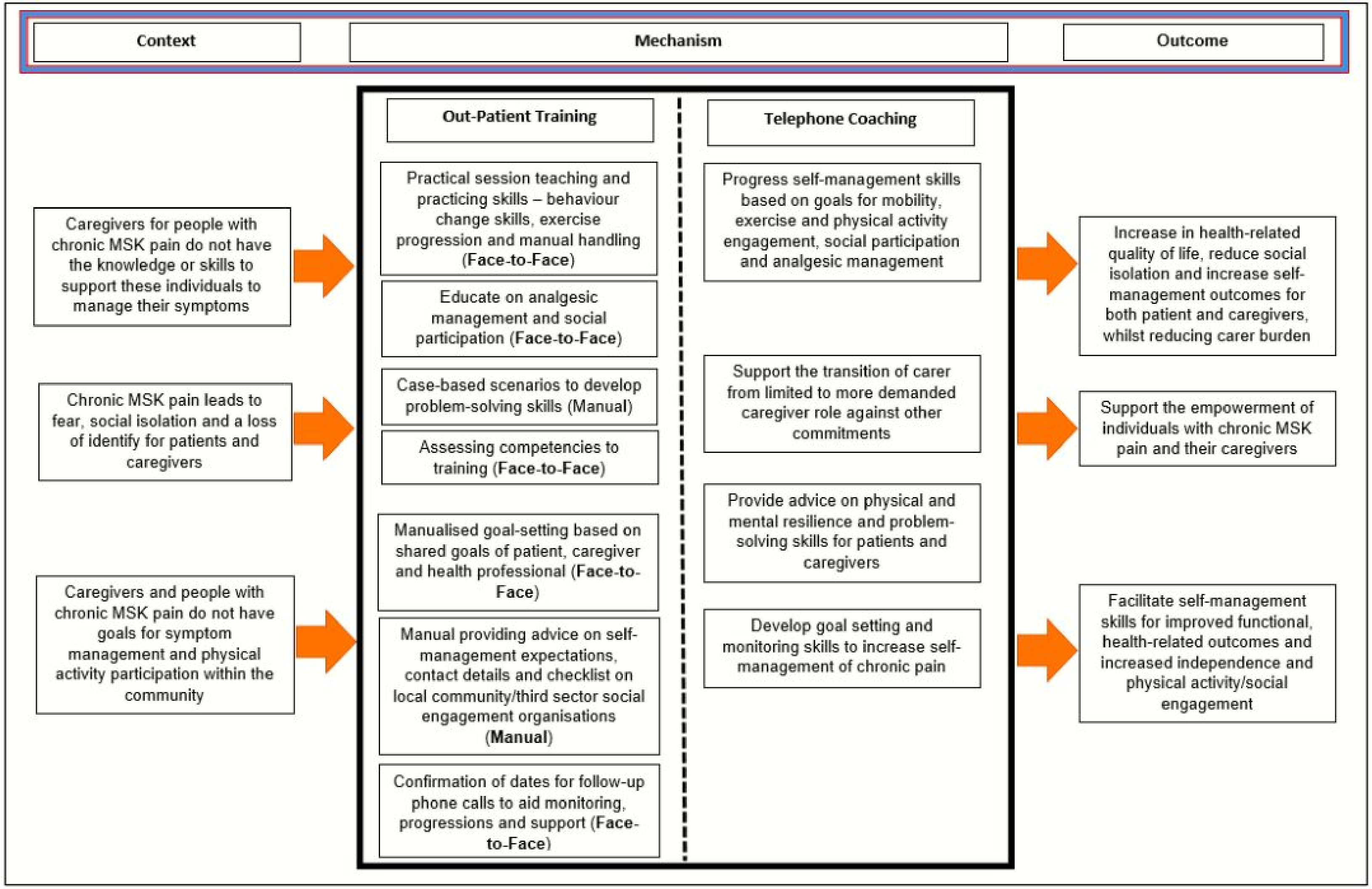

Figure 3: Figure illustrating the JOINT SUPPORT intervention logic model.

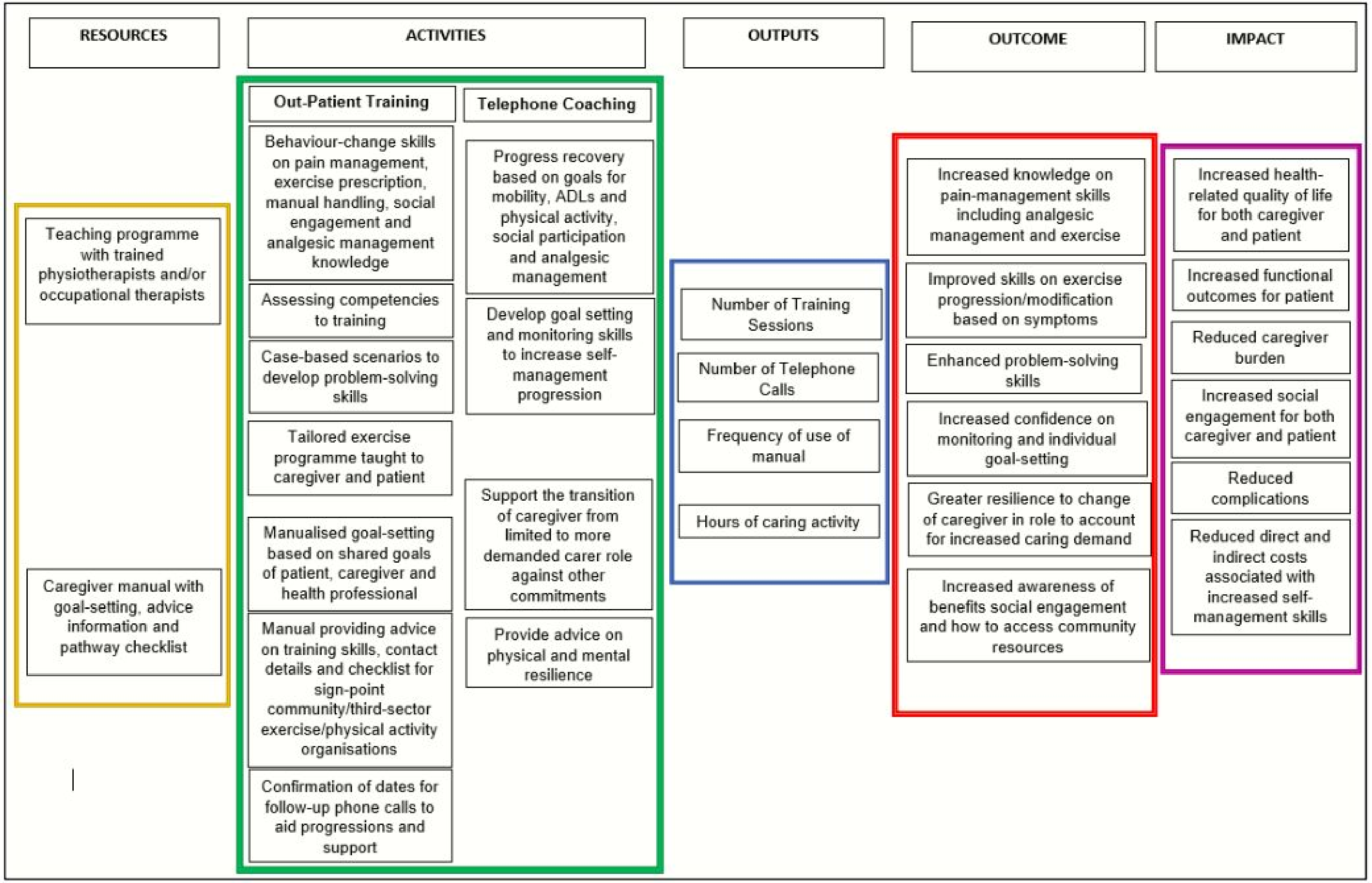

For peer review only - http://bmjopen.bmj.com/site/about/guidelines.xhtml

Figure 4: Participant timeline illustrating schedule of enrolment, interventions, and assessments.

TIMEPOINT	Screening and Initial Contact	Baseline<br>Aim up to 14 days post-first contact	Randomisation	Group-based Session<br>Weeks 1 to 5	Post- session call<br>Aim up to 6 weeks post-group-based session	Follow-Up<br>3 months from post-randomisation (+/- 3 weeks)
ENROLMENT						
Initial approach	■	■				
Informed consent		■				
Randomisation			■			
INTERVENTIONS						
Experimental (Usual care PLUS JOINT SUPPORT)			■	■	■	
Control (Usual Care)			■	■		
ASSESSMENTS						
Screening Logs	■	■				
Adverse event reporting		■	■	■	■	■
Date of First Appointment		■				
Patient age		■				
Patient gender		■				
Patient ethnicity		■				
Patient height		■				
Patient weight		■				
Patient past medical history		■				
Patient occupational status		■				
Patient residential status		■				■
Patient EQ-5D-5L		■				■
Patient MSK-HQ		■				■
Patient GSE		■				■
Patient NRS Pain		■				■
Patient NRS Fatigue		■				■
Patient CES-D		■				■
Patient health utilization questionnaire		■				■
Caregiver EQ-5D-5L		■				■

Caregiver CES-D						
Caregiver Zarit Burden Questionnaire						
Caregiver Leisure Time Satisfaction Questionnaire						
Caregiver health utilisation questionnaire						
Caregiver age						
Caregiver gender						
Caregiver ethnicity						
Caregiver Past Medical History						
Caregiver caregiving experience (duration/caregiver for other person)						
Caregiver residential status to patient						
Caregiver occupational status						
Working or not						
HCP Usual Care Logs						
Joint Support Intervention Logs						
Patient complications/adverse events						
Caregiver Intervention Home Logs						
Caregiver Acceptability Intervention						
Patient-Caregiver Semi Structured Interviews						
HCP Semi Structured Interviews						

CES-D – Centre for Epidemiologic Studies Depression Scale (CES-D); GSE - General self-efficacy scale; HCP – Health Care Professional; MSK-HQ – musculoskeletal health questionnaire

For peer review only

BMJ Open

Informal caregiver training for people with chronic pain in musculoskeletal services (JOINT SUPPORT): protocol for a feasibility randomised controlled trial

Journal:	*BMJ Open*
Manuscript ID	bmjopen-2022-070865.R1
Article Type:	Protocol
Date Submitted by the Author:	09-Jan-2023

Complete List of Authors:	Smith, Toby; University of Warwick; University of East Anglia<br>Smith, Toby; University of East Anglia, Faculty of Medicine and Health Sciences; University of Oxford, Nuffield Department of Orthopaedics, Rheumatology and Musculoskeletal Sciences<br>Khoury, Reema; University of East Anglia, Faculty of Medicine and Health Sciences<br>Ashford, Polly-Anna; University of East Anglia, Faculty of Medicine and Health Sciences<br>Hanson, Sarah; University of East Anglia, Faculty of Medicine and Health Sciences<br>Welsh, Allie; University of East Anglia, School of Education<br>Clark, Allan; University of East Anglia, Faculty of Medicine and Health Sciences<br>Dures, Emma; University of the West of England, School of Health and Social Wellbeing; Bristol Royal Infirmary, Academic Rheumatology<br>Adams, Jo; University of Southampton, School of Health Sciences; University of East Anglia, Faculty of Medicine and Health Sciences

**Primary Subject Heading**:	Rehabilitation medicine
Secondary Subject Heading:	Rheumatology
Keywords:	PAIN MANAGEMENT, REHABILITATION MEDICINE, RHEUMATOLOGY

SCHOLARONE™
Manuscripts

BMJ OPEN

TITLE PAGE

Title:

Informal caregiver training for people with chronic pain in musculoskeletal services (JOINT SUPPORT): protocol for a feasibility randomised controlled trial

Authors:

Smith TO,[1,2] Khoury R,[2] Ashford P,[2] Hanson S,[2] Welsh A,[3] Clark A,[2] Dures E,[4,5] Adams J[2,6]

Affiliations:

1. Warwick Medical School, University of Warwick, Coventry, UK
2. Faculty of Medicine and Health Sciences, University of East Anglia, Norwich, UK
3. School of Education, University of East Anglia, Norwich, UK
4. Academic Rheumatology, Bristol Royal Infirmary, Bristol, UK
5. School of Health and Social Wellbeing, University of the West of England, Bristol, UK
6. School of Health Sciences, University of Southampton, Southampton, UK

Corresponding Author:

Professor Toby Smith, Warwick Clinical Trials Unit, Warwick Medical School, University of Warwick, Coventry, CV4 7AL, UK. Email: toby.o.smith@warwick.ac.uk

Word Count: Abstract: 298; Manuscript: 4341

For peer review only

ABSTRACT

Introduction: Chronic musculoskeletal (bone, joint or muscle) pain is disabling. People with it frequently have difficulties in managing everyday activities. Individuals may rely on family members or friends to support them. These people are known as informal caregivers. No interventions have previously addressed the health needs of people with chronic musculoskeletal pain and their caregivers. In response, the JOINT SUPPORT programme was developed. In this study, we will assess the feasibility and acceptability of conducting a pragmatic, multi-centre randomised controlled trial (RCT) to test the clinical and cost-effectiveness of the JOINT SUPPORT programme to support these individuals.

Methods and Analysis: This will be a mixed-methods feasibility RCT. We will recruit 80 patients with chronic musculoskeletal pain with their informal caregivers. Patients will be randomised to usual National Health Service (NHS) care *OR* usual NHS care plus a caregiver-patient dyad training programme (JOINT SUPPORT). This programme comprises of five, one-hour, group-based sessions for patients and caregivers, delivered by trained physiotherapists or occupational therapists. It includes developing skills in: understanding pain, pacing, graded activity, fear avoidance and goal-setting, understanding benefits of physical activity and skills in medication management. This will be re-enforced with a workbook. After the group-based sessions, patients and caregivers will be supported through three telephone sessions with a therapist. Data collected at baseline and three months will include: screening logs, intervention logs, fidelity checklists and clinical outcomes on quality of life, physical and emotional outcomes, adverse events and resource use. Qualitative research with 24 patient-caregiver dyads and 12 healthcare professionals will explore the acceptability of trial processes. Stop-go criteria will inform the progression to a full trial.

Ethics and Dissemination: Ethical approval was obtained on 22th February 2022 (National Research Ethics Committee Number: 22/NW/0015). Results will be reported at conferences, peer-review publications and across social media channels.

Trial Registration Number: ISRCTN registration: ISRCTN78169443

Status: Trial recruitment is on-going and the study is expected to be completed by June 2024.

Keywords: Pain; Caregiver: Dyad; RCT; Process Evaluation; Rehabilitation

For peer review only

STRENGTHS AND LIMITATIONS OF THIS STUDY

1. JOINT SUPPORT is a pragmatic, multicentre, feasibility RCT.
2. This study will determine if it is feasible to conduct an RCT to assess the effectiveness of a caregiving intervention for people with chronic musculoskeletal pain.
3. The sample size is sufficient to inform decision-making on the design of a larger full-trial.
4. The embedded qualitative study will explore experiences and views on of the study design and intervention.
5. The relative small sample size increases the risk of higher potential loss to follow-up.

INTRODUCTION

Chronic musculoskeletal (bone, joint or muscle) pain is disabling. It is seen in all ages of people.[1] It encompasses conditions such as low back pain, neck pain, fibromyalgia, osteoarthritis, pain from fractures or other rheumatological diseases. Many people have more than one body region affected.[2] It affects approximately 17 million people in the UK, with 9.1 million people living in England with long-term back pain.[3] NHS costs to treat musculoskeletal diseases are in excess of £5 billion per year.[3]

People with chronic musculoskeletal pain frequently have difficulties in managing their symptoms and everyday activities to maintain independence and quality of life.[4] To assist with symptoms, they often access support. This may include help in tasks such as: washing and dressing, preparing meals and assistance in feeding, housework or shopping.[5,6] This caregiving may be formal or informal. Formal care is defined as the provision of care by someone who is paid. Informal care is provided without a direct payment. This is often given by a spouse or partner, family members and/or friends.[7]

Being a caregiver for someone with chronic musculoskeletal pain can be a physical and emotional burden.[8] Becoming a caregiver in this situation can significantly change the dynamic of a relationship with feelings of burden which can led to resentment,[9] changing roles and identities not only between the patient-caregiver dyad but also impacts on family, social and occupational life, and fear and concern regarding the future and how chronic pain may impact on the individual they support, over-time.[9,10] Usual NHS care is focused on the patient, providing interventions to support the long-term management of pain and disability. These are either through structured programmes such as the ESCAPE-Pain programme,[11] or non-structured guidelines incorporating elements of education, exercise, pain relief and psychological interventions.[12-14] In both instances, none of these approaches has included caregiver interventions to support symptom management.

No caregiving interventions have existed to address the health and social needs of people with chronic musculoskeletal pain or to support caregivers. In response, the research team developed the JOINT SUPPORT programme. This is aimed to improve patient symptom management by educating, supporting and empowering caregivers to optimise the support they give their family member or friend with chronic musculoskeletal pain.

Caregiver interventions, in principle, improve the health and wellbeing of patients and their caregivers through better self-management skills.[9] This is important as living with pain is associated with a negative health status, including reduced independence, social isolation and loneliness, obesity and comorbidities associated with physical inactivity such as type two diabetes, depression and cardiovascular disease.[15-17] Improving the skills, capability, motivation, confidence and knowledge so that people with chronic musculoskeletal pain can better manage their symptoms could reduce demand on NHS services through improved self-management and wider health improvement.

AIM AND OBJECTIVES

AIM: To assess the feasibility of conducting a pragmatic, multi-centre randomised controlled trial (RCT) to test the clinical and cost-effectiveness of an informal caregiver training programme to support people with chronic musculoskeletal pain.

OBJECTIVES:

The main objectives are to assess the:

- Feasibility of recruiting eligible patient and caregiver dyads in the NHS.
- Acceptability of the JOINT SUPPORT programme to caregivers, patients and healthcare professionals.
- Fidelity of delivery of the JOINT SUPPORT programme by healthcare professionals.
- Ability of caregivers to deliver components of the JOINT SUPPORT programme with fidelity, confidence and competency to patients at home.
- Acceptability of caregiving dyad randomisation for patients, caregivers and healthcare professionals.
- Risk of intervention contamination when experimental and control groups are delivered in the same setting.

The secondary objectives are to assess the:

- Completeness of outcome measure data.
- Signal of the clinical effectiveness of the JOINT SUPPORT programme.

METHODS AND ANALYSIS

This trial builds on previously performed caregiver intervention methods undertaken by the research team [18].

Trial Design

A mixed-methods feasibility study comprising of a parallel, multicentre, pragmatic RCT and embedded qualitative study. **Figure 1** illustrates the study flow.

Prior to commencing, all participating site team members will be taught the skills and knowledge required to fulfil their delegated role in the trial. They will have completed training, including Good Clinical Practice (GCP)[19] if required. Their participation and competence in delegated roles will be recorded in a site delegation log.

Eligibility

We will include:

- Patients aged 18 years and over with a history (six weeks or more) of pain from a musculoskeletal (bone, joint or muscle) origin who have a current referral to, or who are attending physiotherapy, rheumatology, orthopaedic, occupational therapy or pain management services.
- Patients able to nominate an informal caregiver. An informal caregiver is defined as someone who has done or is expected to provide unpaid care, assistance, support or supervision in activities of daily living for at least three hours per week over two or more personal contacts.

For peer review only

- Patients and caregivers willing and able to provide consent.
- Patients and caregivers who can engage in a group-based intervention currently delivered in English.

If the participating hospital appointment is routinely a virtual appointment rather than in-person, patient and caregiver participants must have access to a device which can receive this If a patient has multiple caregivers, a single, nominated by the patient, 'principal' caregiver will be elected.

We will exclude:

- Patients or caregivers with acute (requiring hospitalisation) or terminal illness (life expectancy < six weeks) which would make participation in the rehabilitation strategies contraindicated and/or impractical.

Recruitment

Patients will be identified and approached during rheumatology, orthopaedic, pain management, occupational therapy or physiotherapy out-patient appointments. A member of the clinical team will screen participants against the eligibility criteria. This will be recorded on the site screening log. For eligible participants, a member of the site clinical team will briefly outline the study and offer a Recruitment Information Pack. The pack includes: covering letter, Participant Information Sheet (PIS, separate for patient and caregiver), Consent Form (patient and caregiver), Baseline Questionnaires (separate for patient and caregiver), Participant Contact Details Form (separate for patient and caregiver) and Demographic Characteristics Form (patient and caregiver) with a pre-paid envelope. With this information, potential participants will be asked to consider participation. If a caregiver is present with the patient at the appointment, they will be provided with the caregiver PIS. If not, the patient will be provided with this to give to the caregiver. The potential patient and caregiver participant will be asked to read this information. After a minimum of 24 hours, each participant provided with the Recruitment Information Pack will be telephoned or video-called by the site team member and provided with an opportunity to ask questions. They will be informed that if they would like to participate, they should complete the Consent Form, and then (sequentially) Participant Contact Details Form (patient and caregiver) and Demographic Characteristics Form (patient and caregiver) and Baseline Questionnaire (patient and caregiver) and to return them using the pre-paid envelope to the Central Trial Team, who will then randomise the participant-dyad and notify the site team of both enrolment and group allocation. We will aim to recruit potential patient and caregiver participants within 14 days of the initial contact.

All screening activity will be recorded by a member of the trained local site team using the trial's screening log. Eligible participants who are approached but who do not wish to participate will be anonymously recorded as part of a screening log, providing information on: age, gender, date of appointment and type of musculoskeletal pathology.

Both caregiver and patient consent will be required.

Randomisation, blinding and allocation concealment

Randomisation will take place once patient and caregiver participant consent forms and baseline questionnaire packs have been completed, received and checked by a member of the Central Trial Team at Norwich Clinical Trials Unit (NCTU). The randomisation scheme will be generated by the NCTU

For peer review only

Trial Manager and allocated group will be notified by email to the site trial team. Randomisation will be stratified at the individual dyad level (approximately 2:1) by:

- Site
- Age of patient (< or > and equal to 65 years).

The allocation is computer generated so will not be known prior to randomisation. Allocation is concealed prior to randomisation to prevent selection (or treatment allocation) bias.

Due to the participatory nature of rehabilitation, it will not be possible to blind patients, caregivers or treating healthcare professionals to group allocation after randomisation. The trial PIS has presented intervention equipoise. Therefore, whilst not possible to blind, we have aimed to limit expectation bias through careful selection of PIS wording for participants.

Intervention

Usual Care

This will be National Health Service (NHS) treatment as usual (TAU). Participant dyads randomised to this group will receive standard NHS care delivered by their department e.g., physiotherapy, rheumatology, orthopaedic, occupational therapy or pain management services. This consists of patient-focused treatments which may include exercise, medication prescription and advice/education.[11-14] This may be face-to-face or through video consultation approaches depending on local NHS service provision. There is no routine 'training' element or hands-on skills or formal support for caregivers. Patients and their caregivers will not receive the JOINT SUPPORT programme or any caregiver training.

Treatment logs will be used to record the components of standard care. This will be completed by the clinical team to determine what interventions are received by the patient whilst they are under the care of that service.

Experimental Intervention

The JOINT SUPPORT programme was developed from the evidence-base[9] and following patient, caregiver and stakeholder input. The programme is developed and informed by Social Cognitive Theory.[20] This is an explanation for behaviour in which individual learning happens in a social context, where people can put vicarious learning into practice, and learn together and from each other in the process. The three goals of the intervention are outlined below using a CONTEXT-MECHANISM-OUTCOME framework (**Figure 2**)[21] and logic model (**Figure 3**).

To improve caregiver knowledge, capability and skills by identifying and practising individualised chronic pain management strategies and physical activity participation in out-patient (or video consultation) settings that can be used for people with MSK pain

Caregivers of people with chronic musculoskeletal pain (CONTEXT) need the skills, capability opportunity and knowledge (MECHANISM) to be able to support and guide behaviour change for both patients and caregivers to increase health-related quality of life (HRQoL), reduce social isolation and improve self-management outcomes for patients (OUTCOME).

For peer review only

To reduce caregiver fear of movement and isolation when providing care for people with chronic pain

Chronic musculoskeletal pain leads to fear of movement, isolation and a loss of identity for caregivers (CONTEXT) requiring a re-evaluation of their role and identity (MECHANISM) to be able to support people with chronic musculoskeletal pain and promote good caregiver health and wellbeing (OUTCOME).

Support patients and caregivers to increase capability, set targets and monitor goals to facilitate patients improved symptom management

Patients with chronic musculoskeletal pain in an out-patient setting (CONTEXT) will be supported by caregivers to set personalised shared goals which they can realistically meet (MECHANISM) to facilitate improved functional, health-related outcomes and increased independence (OUTCOME).

Participants randomised to the JOINT SUPPORT programme will receive usual NHS treatment (control group intervention as detailed below) PLUS five, one-hour, group-based training sessions, delivered by a study-trained physiotherapist or occupational therapist. The intervention will be delivered through two possible approaches.

Approach 1: Face-to-face delivery which will be group-based, consisting of a target three to five dyads in each class. They will be delivered in an out-patient setting provided to both participants in the dyad by a study-trained physiotherapist or occupational therapist.

Approach 2: Video consultation if face-to-face consultations are not permitted within a participating NHS organisation, group-based video consultation sessions using an NHS authorised platform will be used to deliver the content of the JOINT SUPPORT programme as per the face-to-face delivery approach.

If there is a change in service delivery once a participant-dyad has started their JOINT SUPPORT programme, switching from Approach 1 to Approach 2 will be permitted, with the effects explored in the qualitative sub-study.

JOINT SUPPORT Training Programme

The first session will aim to start within six weeks from consent. Sessions will be provided to both patient and caregiver participants by either a physiotherapist or occupational therapist. Irrespective of delivery method, each session will be up to 60 minutes. Throughout the five sessions, the JOINT SUPPORT healthcare professional will monitor the patient-caregiver competencies, providing continual feedback and critique to support the training. There will be a cognitive-behavioural approach used throughout the sessions, underpinned by social cognitive theory where dyads will be facilitated by a JOINT SUPPORT healthcare professional on the following topics:

Session 1:

- Understanding pain, caregiving and how pain affects the caregiving dyad
- Introduction and explanation of the JOINT SUPPORT Workbook, highlighting material on pain and effects on the dyad

Session 2:

- Pacing and graded activity

For peer review only

- Goal setting
- JOINT SUPPORT Workbook - highlighting material on goal-setting and problem-solving

Session 3:

- Benefits of physical activity (reducing deconditioning, healthy ageing, physical and psychological health)
- Fear avoidance
- JOINT SUPPORT Workbook - highlighting material on physical activity and fear avoidance

Session 4:

- Medication use and management
- JOINT SUPPORT Workbook - highlighting material on medication use and management

Session 5:

- Working through case-study scenarios to re-enforce knowledge and critique competencies on JOINT SUPPORT skills
- JOINT SUPPORT Workbook - highlighting material on case-scenarios and long-term goal setting
- Confirmation of dates for JOINT SUPPORT Telephone Booster calls

JOINT SUPPORT Telephone Booster Calls

Following the five group-sessions, a JOINT SUPPORT healthcare professional will telephone each caregiver and patient as a dyad during Week 1, 3 and 6 post-group session discharge. Each call is expected to take approximately 20 minutes. Both the caregiver and patient participant should be in the same room during these calls. Topics covered in each call will include:

- Recovery progress and current status based on patient-caregiver shared goals
- Discussion on JOINT SUPPORT Workbook use and progress including goal-setting sheets
- Support to create collaborative goals and positive reinforcement for continued recovery

JOINT SUPPORT Healthcare Professional Training

Designated healthcare professionals delivering the experimental intervention will attend a one-day face-to-face course where, at the local participating site, they will be taught the JOINT SUPPORT intervention by a member of the research team. In addition, to assess the fidelity to the experimental intervention, the trial team will undertake a debriefing telephone/video call with healthcare professional after they have delivered their first JOINT SUPPORT session.

Contamination

Participants will only receive their allocated intervention. During follow-up, participants may require further interventions as part of their recovery as per routine NHS practice. Further clinical interventions will be permitted for participants without having to withdraw from the trial, but these will be recorded by the Central Trial Team. To assess for the risk of crossover between groups, we will closely monitor case report forms (CRFs) and data pertaining to intervention delivery.

Co-interventions

This is a pragmatic study and patient-caregiver dyads in either group will not be asked to desist from receiving other forms of treatment during the study such as continuing rehabilitation, general

For peer review only

practitioner (GP) consultations, medication changes or alternative treatments as required. Use of these treatments will be recorded through CRFs.

Quality Assessment

The trial will be monitored and audited in accordance with the current approved protocol, principles of GCP,[19] relevant regulations and Standard Operating Procedures (SOPs).

A quality control programme will be adopted to ensure protocol and intervention fidelity. We will collect data on what components of the interventions (control and experimental) were delivered. This is in respect of intervention parameters including: content, mode of delivery, staff delivered, frequency, timing of delivery and variation/deviations from the protocol. These will be collected through intervention logs and relevant CRFs. Quality Assurance (QA) checks through site visits will be conducted at Month 1 from first randomisation (+/- three weeks for each) at each site. These visits may be in-person or virtual. If there are concerns in relation to any aspect of the site visit, repeat visits with training may be undertaken to improve protocol compliance.

Assessment

The data collection schedule is summarised in **Table 1**.

To answer our feasibility objectives, we will assess:

- Recruitment feasibility – by screening log data: number of potential patients and caregivers screened, assessed for eligibility; including reasons for exclusion/non-participation, and consented.
- Intervention acceptability – by conducting qualitative interviews with participants; recording study attrition at the intervention phase; analysing acceptability questionnaires for patients, caregivers and healthcare professionals.
- Intervention fidelity (healthcare professionals) – by analysis and reporting on intervention log checklist data on: intervention timing, duration, frequency, timing of intervention (during or after 'active' course of treatment); QA monitoring visit checklists; qualitative interviews.
- Intervention fidelity (caregivers) – by analysis and reporting on caregiver intervention logs; qualitative interviews.
- Randomisation acceptability – by analysis and reporting on screening logs, eligibility assessment logs and consent forms; participant attrition; qualitative interviews.
- Contamination risk - by analysis and reporting on intervention log data including: experimental and control intervention records; QA monitoring visit checklists; delegation logs; qualitative interviews (patient, caregiver and healthcare professional).

Secondary outcomes

- Outcome data completeness – by analysis and reporting on completion rates (baseline and three months post- randomisation) of: *Patient Participants*: Musculoskeletal-Health Questionnaire (MSK-HQ);[22] numerical rating scale for pain and fatigue;[23] self-efficacy, assessed using the General Self-Efficacy Scale (GSE);[24] psychological distress (depression), assessed using the Center for Epidemiologic Studies Depression Scale (CES-

For peer review only

D);[25] HRQoL assessed using the EQ-5D-5L;[26] self-reported health resource use questionnaire; adverse events. *Caregiver Participants*: EQ-5D-5L;[26] CES-D;[25] the Zarit Burden Interview Scale – 12-item version;[27] Leisure Time Satisfaction questionnaire (LTS);[28] self-reported health resource use questionnaire; adverse events.

For peer review only

Table 1: Participant timeline illustrating schedule of enrolment, interventions, and assessments.

TIMEPOINT	Screening and Initial Contact	Baseline<br>Aim up to 14 days post-first contact	Randomisation	Group-based Session<br>Weeks 1 to 5	Post- session call<br>Aim up to 6 weeks post-group-based session	Follow-Up<br>3 months from post-randomisation (+/- 3 weeks)
ENROLMENT						
Initial approach	■	■				
Informed consent		■				
Randomisation			■			
INTERVENTIONS						
Experimental (Usual care PLUS JOINT SUPPORT)			■	■	■	
Control (Usual Care)			■	■		
ASSESSMENTS						
Screening Logs	■	■				
Adverse event reporting		■	■	■	■	■
Date of First Appointment		■				
Patient age		■				
Patient gender		■				
Patient ethnicity		■				
Patient height		■				
Patient weight		■				
Patient past medical history		■				
Patient occupational status		■				
Patient residential status		■				■
Patient EQ-5D-5L		■				■
Patient MSK-HQ		■				■
Patient GSE		■				■
Patient NRS Pain		■				■
Patient NRS Fatigue		■				■
Patient CES-D		■				■
Patient health utilisation questionnaire		■				■
Caregiver EQ-5D-5L		■				■

For peer review only

Caregiver CES-D						
Caregiver Zarit Burden Questionnaire						
Caregiver Leisure Time Satisfaction Questionnaire						
Caregiver health utilisation questionnaire						
Caregiver age						
Caregiver gender						
Caregiver ethnicity						
Caregiver Past Medical History						
Caregiver caregiving experience (duration/caregiver for another person)						
Caregiver residential status to patient						
Caregiver occupational status						
Working or not						
HCP Usual Care Logs						
Joint Support Intervention Logs						
Patient complications/adverse events						
Caregiver Intervention Home Logs						
Caregiver Acceptability Intervention						
Patient-Caregiver Semi Structured Interviews						
HCP Semi Structured Interviews						

CES-D – Centre for Epidemiologic Studies Depression Scale (CES-D); GSE - General self-efficacy scale; HCP – Healthcare Professional; MSK-HQ – musculoskeletal health questionnaire

For peer review only

- Signal of effectiveness – by calculating and reporting effect size measurement of the above outcome measures.

These measures were selected due to their robust psychometric properties and acceptable patient and caregiver burden as confirmed by our study Patient Representatives/clinician feedback. They reflect relevant core outcome sets including: lower limb osteoarthritis,[29] low back pain[30] and general musculoskeletal disease.[31] Caregiver outcomes mirror the domains of importance reported through previous qualitative findings.[9]

Baseline assessment will be undertaken after patient and caregiver participants have signed the consent form, prior to randomisation. Patient and caregiver participants will be provided with a paper-based questionnaire to complete in their Recruitment Information Pack. Baseline data collected will include: (for patient and caregiver): age, sex, ethnicity, occupational status (current or past if retired), medical co-morbidities, presenting musculoskeletal pathology(ies); and for patient, duration of symptoms relationship of caregiver to patient; and for caregiver: duration of caregiving, whether caregiver for another person, whether lives with patient.

At three months post-randomisation, patient and caregiver participants will be sent a postal questionnaire. A three-month follow-up period will provide an indication on follow-up completion and usability of the outcome instruments to answer a trial secondary outcome measure. Participants will be asked to complete and return this to the Central Trial Team using a pre-paid envelope. If participants do not return these questionnaires within two weeks of initial posting, the Central Trial Team will telephone the participants (caregiver and/or care-recipient) to offer the option of completing the questionnaires over the telephone or to send a further questionnaire pack.

Data Analysis

Sample Size

In total, 80 participant dyads (80 patients/80 caregivers) will be recruited. This sample size will be sufficient to answer our feasibility objectives and assess the *a priori* progression criteria (**Table 2**).[32] This number will also allow each site to test the JOINT SUPPORT programme in at least two complete cycles. This sample size follows recommendations[33] for designing feasibility trials that aim to detect a small-medium standardised effect size (where the definitive trial will be designed with 80% power and two-sided 5% significance).[32,34]

Table 2: Feasibility study stop-go, traffic light, progression criteria

	Green (Go)	Amber (Amend)	Red (Stop)
**Recruitment**	>30% of patients screened across the sites in 12 months would be eligible	20% to 30% would be eligible	<20% would be eligible
**Intervention Fidelity (Healthcare Professionals)**	>70% of participant-dyads compliant with their allocated intervention (5 face-to-face sessions and booster phone call) as randomised	50% to 70% received intervention as randomised	<50% received intervention as randomised

For peer review only

**Intervention Fidelity (Caregivers)**	>90% of participants adopted elements of JOINT SUPPORT programme post-last session	60% to 90% adopted JOINT SUPPORT post-last session	<60% adopted elements post-last session
**Randomisation Acceptability**	>40% of eligible participants consent to be randomised	20%-40% would be randomised	<20% would be randomised
**Contamination**	<5% of participants in either group received majority of their allocated treatment cross-over	5%-10% of participants cross-over	>10% of participants cross-over
**Data Collection Completion**	<15% missing outcome questionnaires for whatever reason at 3-month data collection	15%-30% missing questionnaires	>30% missing questionnaires

Analysis

Consent rates, recruitment rates, attrition, missing data rates and intervention fidelity will be reported as proportions with 95% confidence intervals (CIs). The analysis of clinical outcome measures will be descriptive, reported as means and standard deviations or medians and interquartile ranges if not normally distributed for continuous outcomes and numbers and percentages for binary and categorical variables. Between-group mean differences will be reported together with 95% CIs. No formal statistical testing will be undertaken.

Health Economic

Data on health care utilisation will be collected but not analysed. To answer the feasibility questions related to the health economic perspectives, we will test the completion of the health resource use questionnaire and will present the data descriptively.

Progression Criteria

A 'traffic light' system (**Table 2**) will be used as a guide for progression to a definitive trial.[35] If any criteria are not met, they will be discussed by the Trial Oversight Committee (TOC) to decide if a definitive trial is feasible with, or without, refinements.

Qualitative study

The embedded qualitative investigation will assess intervention and study design acceptability for patients, caregivers and healthcare professionals.

Eligibility

The embedded qualitative study is optional for participants. Patient and caregivers will be able to express an interest to be approached regarding the qualitative sub-study on the main study consent form at the beginning of the study. The sample will consist of patient-caregiver dyads and physiotherapists and occupational therapists who have delivered the JOINT SUPPORT intervention.

For peer review only

Dyads who have agreed to be contacted for an interview will be purposively sampled to ensure a maximum variation in: age, gender, presenting musculoskeletal pathology, ethnicity, duration of disability, severity of disability (as measured by the baseline MSK-HQ,[22] relationship of caregiver to patient and duration the caregiver has been caring for the patient. Our intention is for caregivers and patients to be interviewed separately to enable them to more freely express their views but we will interview as a dyad if this is expressly requested.

We anticipate around twenty-four interviews will be conducted, involving approximately 12 patients/12 caregivers from the JOINT SUPPORT programme and approximately eight caregivers/eight patients from the TAU group across the sites. Based on our previous research,[9] this sample size should ensure a range of different viewpoints to reach information power[36] to answer our feasibility questions.

The healthcare professionals delivering the JOINT SUPPORT intervention will be invited to participate in the voluntary interviews within three months of completing one participant through the JOINT SUPPORT programme. A minimum target of one physiotherapist and one occupational therapist who delivered the intervention will be invited to be interviewed from each site (eight participants in total). This will provide a range of contexts from different professional backgrounds. The healthcare professionals will be sent a PIS outlining the objectives and processes involved with this optional sub-study. They will be asked to contact the research team by email if they are interested in participating. Those who express an interest will be contacted to arrange a mutually convenient time to conduct the interview.

Data Collection

Interviews will aim to be conducted up to six weeks post-intervention via telephone or video call, at a time convenient to dyads and healthcare professionals. This allows exploration and reflection of the patients' and caregivers' study experience once the intervention has ended in a reasonable recall period. Interviews will be semi-structured, following an open-ended question schedule, with a maximum duration of 60 minutes. Open-ended questions will explore the acceptability of the research based on Sekhon et al's[37] acceptability framework including the values of the intervention, burden, perceived effectiveness, self-efficacy and suggestions for improvement from their perspectives. Interviews will be conducted by a qualitative researcher (AW), closely supervised by a senior qualitative researcher (SH).

Prior to conducting the interview, consent will be obtained and audio recorded from the participant. The interview will then follow a semi-structured, open-ended question schedule (**Table 3; Table 4**). Broadly, participants will be asked their attitudes towards topics including the:

- Timing, frequency, duration and content of the JOINT SUPPORT training programme and telephone booster calls
- Support and training provided for the group-based programme and telephone boosters
- Recording of both components of the JOINT SUPPORT programme
- Logistic of managing the participant flow
- Their views on the recruitment process
- Acceptability of the JOINT SUPPORT programme
- Potential modifications or recommended changes to the JOINT SUPPORT programme
- Perceived effectiveness/helpfulness

For peer review only

Table 3: Interview topic guide for the caregiving dyad interviews

The interview will be structured on the following areas of interest	Sample questions
Introduction	Overall, could you share your experiences of being involved with our research?
Determining participant views of their intervention	First of all, can you talk me through what study treatment you received? (prompt – clarify what was JOINT SUPPORT and what was usual care/non-study intervention)
The approach and consent process and willingness to be randomised to either group	Can you talk me through how you got into the study? You were allocated to *X* group. What did that feel like? Could we have dealt with that differently?
The acceptability of the care (both groups)	Would you be happy to talk me through your treatment?<br>As *X*'s carer, what was your impression of the care. For both of you, what was helpful and less helpful to your care?
Group-based JOINT SUPPORT programme and telephone booster calls (experimental group)	How far did you find the JOINT SUPPORT programme helpful – for both of you. Can you give specific examples? What didn't work as well?<br>Did you get the telephone calls from the trial team? Can you remember what you talked about? Can you give specific examples of what was helpful, and l helpful? Was there any advice that confused you or you weren't clear about?
What the strengths of the experimental intervention (perceived effectiveness)	What were the most helpful/good-bits of your JOINT SUPPORT intervention? What was good about it. What didn't you like about it?
What the weaknesses of the experimental intervention	What were the less helpful/worse bits of the JOINT SUPPORT intervention?
What modifications they may recommend to interventions received (standard care and experimental groups)	What could we improve? (prompt: What do you think is lacking?)<br>How do you think we could better support you and your carer to support you with chronic pain?
The risk of intervention contamination between the groups	Did you talk to any other patients or caregivers whilst in hospital about the intervention? Was there any discussion between those who received it and did not receive it?
The ease and convenience of the data collection processes (baseline and three months) (all participants)	As you were part of a trial, we had to collect a lot of measurements. Can you talk me through what these were? How easy were they? How convenient were they? Overall, do you have any points to make about the testing?

Applicability of the methods and measures used	How did you manage with the questionnaires we gave you at the start of the study and at the end in the post? Were they easy to complete or do you remember them being a problem?

Table 4: Interview topic guide for the Healthcare Professional Interviews

The interview will be structured on the following areas of interest	Sample questions
Introduction (Perceived effectiveness)	Overall, could you share your experiences of being involved with our research?
The randomised to either group	How did you feel about 50% of the patients not receiving the JOINT SUPPORT intervention but getting normal care? Did this 'sit easy' with you?
The acceptability of the group-based care	How did the delivery of the JOINT SUPPORT group sessions go? How did you work out who would do what? Was there a decision on professional background? Did you feel comfortable teaching all the content? Were any modifications made? How did the patients and caregivers get on with it in your opinion?
JOINT SUPPORT Telephone Calls	How did you feel about doing the telephone calls? Were they helpful for caregivers and patients? Was it feasible to deliver one call to both members of the dyad? Did you make any modifications to the content of the call?
Training on Intervention	Did you feel adequately prepared to deliver the group-based and telephone JOINT SUPPORT interventions? Would you recommend any changes to this? Did you need any additional 'top up' or 'refresher' training sessions?
The risk of intervention contamination between the groups	Do you think you used the JOINT SUPPORT intervention on control or non-trial patients? Did other professionals not in the trial use the intervention? If either occurred, do you think anything could have been done to avoid this?
The ease and convenience of the data collection processes	As you were part of a trial, we had to collect a lot of measurements. How easy were the intervention data collection logs? How convenient were they? What changes would you recommend if any were needed?

For peer review only

Data Analysis

All interviews will be audio-recorded, transcribed and anonymised. After transcription, the audio data will be destroyed. Data will be analysed thematically taking a two-stage approach to understand the important contextual factors that have influenced the implementation of JOINT SUPPORT. We aim to initially analyse all data deductively, guided by the MRC guidance for complex interventions,[38-40] to assess the quality of implementation, clarify the hypothesised causal mechanisms identified in our logic model (for example, goal setting in the training and the support provided by the telephone coaching), identify contextual factors associated with variation in outcomes and how the intervention might be optimised for acceptability.[37] Data will then be analysed more inductively and more broadly. This will include critiquing the conceptual approach of JOINT SUPPORT, understanding any unintended consequences and reflections on the JOINT SUPPORT intervention from the healthcare professional, patient and caregiver perspective.

Trial Status

The trial is funded for 24 months commencing in April 2022. Recruitment is expected to be complete by June 2023 with the final follow-up visit completed by November 2023. The trial will be completed by April 2024.

Patient and Public Involvement

Patients have been involved with the study development from inception. Their involvement will continue throughout the trial. A patient-dyad member will attend TOC meetings. They will provide insights into the trial conduct, particularly on data collection processes. They will also help interpret the findings and the dissemination phase.

ETHICS AND DISSEMINATION

All data will be processed according to the Data Protection Act.[41] All documents will be stored safely in confidential conditions. Trial-specific documents, except for the signed consent form and follow-up contact details, will refer to the participant with a unique study participant number, not by name. Participant identifiable data will be stored separately from trial data. All trial data will be stored securely in offices or online in secure trial databases, only accessible by the Central Trial Team and authorised personnel. Participant withdrawal is permitted at any point post-enrolment. This will be recorded on a trial CRF, including reasons for withdrawal. Adverse events and Serious Adverse Events will be recorded and reported in-accordance to ICH GCP.[19]

The results of the trial will be reported using the CONSORT guidelines[42] including the relevant extensions for non-pharmacological interventions and for pilot and feasibility studies. The intervention will be reported according to the TIDIER guidelines.[43] This will be presented to academic, clinical and patient and public audiences. To ensure that outputs are accessible to diverse stakeholder groups, we will develop result materials across our research team including patient representatives.

For peer review only

For peer review only

ACKNOWLKEDGEMENTS AND DECLARATIONS

JOINT SUPPORT Collaborators: Mrs Coralie Crowther (Norfolk, UK), Mrs Shelia Macaulay-Cripps (Norwich, Norfolk), Mr John Macaulay-Cripps (Norwich, Norfolk), Jessica Pawson, Joanna Fletcher (Principle Investigator) and Catherine Hilton (Barts Health NHS Trust, London), Gregory Booth (Royal National Orthopaedic Hospital NHS, Trust, London - Principle Investigator), Gareth Stephens (The Royal Orthopaedic Hospital NHS Foundation Trust, Birmingham – Principle Investigator), Caroline Swain (The Royal Orthopaedic Hospital NHS Foundation Trust, Birmingham), Sarah Rich (The Royal Orthopaedic Hospital NHS Foundation Trust, Birmingham) and Chris Drake (Principle Investigator) and Tom Shelton (Mid-Yorkshire Hospitals NHS Trust, Wakefield).

Trial Oversight Committee Members: Professor Patricia Schofield (Chair) (University of Plymouth), Professor Lindsay Bearne (St George's University, London), Dr Catherine Best (University of Stirling). Trial Oversight Committee Charter can be accessed through reasonable request to the Corresponding Author.

Trial Sponsor: University of East Anglia, Norwich, NR4 7TJ, UK (Email: rin.fmh@uea.ac.uk).

Funding: This study is funded by the National Institute for Health Research (NIHR) Research for Patient Benefit grant (NIHR202723). The views expressed are those of the authors and not necessarily those of the NIHR or the Department of Health and Social Care.

*Neither the funder or sponsor have ultimate authority over the analysis, interpretation, writing of reports or submission of publications.

Protocol Number: Protocol Version 3.1 – Dated 15 September 2022

Contributions: TOS, RK, PAA, SH, AW, AC, ED, JA developed the study concept and design; TOS, ED, JA provide clinical expertise; SH, AW led the qualitative research design; AC led the statistical analysis design; TOS, AC, RK, PAA, SH, ED, JA provided trial design and conduct expertise; TOS, RK, PAA, SH, AW, AC, ED, JA drafted the protocol paper and agreed the submission; TOS acts as guarantor.

Ethical Approvals: Ethical approval was obtained on 22nd February 2022 (National Research Ethics Committee Number: 22/NW/0015).

Trial Registration Number: ISRCTN registration: ISRCTN78169443

Competing Interests: None declared

Patient consent for publication: Not required.

Provenance and Peer-Review: Not commissioned: externally peer-reviewed

Data availability statement: Data will be available on reasonable request. Access to the de-identified dataset for purposes of research other than this study, will be at the discretion of the Chief Investigator, Professor Toby Smith and NCTU. Requests for the de-identified dataset generated during the current study should be made to the Chief Investigator, Professor Toby Smith (email:

For peer review only

toby.o.smith@warwick.ac.uk) or NCTU (email: hiphelper@uea.ac.uk). Professor Toby Smith and NCTU will consider requests once the main results from the study have been published up until 31 June 2027.

For peer review only

FIGURE AND TABLE LEGENDS

Figure 1: Study flow-chart illustrating the participant flow for the JOINT SUPPORT study

Figure 2: Figure illustrating the Context-Mechanism-Outcome framework for the JOINT SUPPORT intervention.

Figure 3: Figure illustrating the JOINT SUPPORT intervention logic model.

Table 1: Feasibility study stop-go, traffic light, progression criteria

Table 2: Interview topic guide for the caregiving dyad interviews

Table 3: Interview topic guide for the Healthcare Professional Interviews

Table 4: Participant timeline illustrating schedule of enrolment, interventions, and assessments.

For peer review only

REFERENCES

1. Smith E, Hoy DG, Cross M, *et al*. The global burden of other musculoskeletal disorders: estimates from the Global Burden of Disease 2010 study. *Ann Rheum Dis* 2014;**73**:1462-9.

2. Raja R, Dube B, Hensor EM, *et al*. The clinical characteristics of older people with chronic multiple-site joint pains and their utilisation of therapeutic interventions: data from a prospective cohort study. *BMC Musculoskelet Disord* 2016;**17**:194.

3. Public Health England. Health Profile for England. Public Health England 2021. Available at: https://fingertips.phe.org.uk/static-reports/health-profile-for-england/hpfe_report.html Accessed on: 06Dec2022

4. Wolff JL, Spillman BC, Freedman VA, Kasper JD. A national profile of family and unpaid caregivers who assist older adults with health care activities. *JAMA Intern Med* 2016;**176**:372-9.

5. Whybrow P, Moffatt S, Kay L, Thompson B, Aspray T, Duncan R. Assessing the need for arthritis training among paid carers in UK residential care homes: A focus group and interview study. *Musculoskeletal Care* 2018;**16**:82-9.

6. Riffin C, Van Ness PH, Wolff JL, Fried T. Family and other unpaid caregivers and older adults with and without dementia and disability. *J Am Geriatr Soc* 2017;**65**:1821-8.

7. Smith TO, Mansfield M, Hanson S, *et al*. Caregiving for older people living with chronic pain: analysis of the English Longitudinal Study of Ageing and Health Survey for England. *Br J Pain* 2022; In Press.

8. Darragh AR, Sommerich CM, Lavender SA, *et al*. Musculoskeletal discomfort, physical demand, and caregiving activities in informal caregivers. *J Appl Gerontol* 2015;**34**:734–60.

9. Smith T, Fletcher J, Lister S. Lived experiences of informal caregivers of people with chronic musculoskeletal pain: a systematic review and meta-ethnography. *Br J Pain* 2021;**15**:187-98.

10. Richardson JC, Ong BN, Sim J. Experiencing chronic widespread pain in a family context: giving and receiving practical and emotional support. *Sociol Health Illn* 2007;**29**:347–65.

11. Hurley MV, Walsh NE, Mitchell HL, *et al*. Clinical effectiveness of a rehabilitation program integrating exercise, self-management, and active coping strategies for chronic knee pain: a cluster randomized trial. *Arthritis Rheum* 2007;**57**:1211-9.

12. NICE. Osteoarthritis: care and management - 2014. Guideline CG 177. Accessed: 06Dec2022. Available at: https://www.nice.org.uk/guidance/cg177

13. NICE. Chronic pain (primary and secondary) in over 16s: assessment of all chronic pain and management of chronic primary pain - 2021. Guideline NG193. Accessed: 06Dec2022. Available at: https://www.nice.org.uk/guidance/ng193

For peer review only

14. NICE. Low back pain and sciatic in over 16s: assessment and management – 2020. Guideline NG59. Accessed: 06Dec2022. Available at: https://www.nice.org.uk/guidance/ng59

15. Katz J, Rosenbloom BN, Fashler S. Chronic pain, psychopathology, and DSM-5 somatic symptom disorder. *Can J Psychiatry* 2015;**60**:160-7.

16. Hassoon A, Bydon M, Kerezoudis P, Maloney PR, Rinaldo L, Yeh HC. Chronic low-back pain in adult with diabetes: NHANES 2009-2010. *J Diabetes Complications* 2017;**31**:38-42.

17. Fayaz A, Ayis S, Panesar SS, Langford RM, Donaldson LJ. Assessing the relationship between chronic pain and cardiovascular disease: A systematic review and meta-analysis. *Scand J Pain* 2016;**13**:76-90.

18. Smith T, Clark L, Khoury R, Man MS, Hanson S, Welsh A, Clark A, Hopewell S, Pfeiffer K, Logan P, Crotty M, Costa M, Lamb SE. A feasibility study to assess the design of a multicentre randomized controlled trial of the clinical and cost-effectiveness of a caregiving intervention for people following hip fracture surgery. *Bone Jt Open* 2021;**2**:909-920.

19. ICH. 2022. ICH Harmonisation for Better Health. Accessed: 06Dec2022. Available at: https://www.ich.org/

20. Bandura A. *Social foundations of thought and action: A social cognitive theory*. Prentice-Hall, Inc, 1986.

21. Pawson R, Tilley N. *Realistic evaluation*. London: SAGE; 1997.

22. Hill JC, Kang S, Benedetto E, *et al*. Development and initial cohort validation of the Arthritis Research UK Musculoskeletal Health Questionnaire (MSK-HQ) for use across musculoskeletal care pathways. *BMJ Open* 2016;**6**:e012331.

23. Farrar JT, Young JP Jr, LaMoreaux L, Werth JL, Poole MR. Clinical importance of changes in chronic pain intensity measured on an 11-point numerical pain rating scale. *Pain* 2001;**94**:149-58.

24. Schwarzer R, Jerusalem M. Generalized Self-Efficacy scale. In J. Weinman, S. Wright, & M. Johnston, *Measures in health psychology: A user's portfolio. Causal and control beliefs* (pp. 35-37). Windsor, UK: NFER-NELSON, 1995.

25. Radloff LS. The CES-D scale: A self-report depression scale for research in the general population. *Appl Psycholog Measurement* 1977;**1**:385-401.

26. EuroQol: EQ-5D. Accessed: 06Dec2022. Available at: http://www.euroqol.org/

27. Hebert R, Bravo G, Preville M. Reliability, validity and reference values of the Zarit Burden Interview for assessing informal caregivers of community-dwelling older persons with dementia. *Canadian J Aging* 2000;**19**:494-507.

28. Stevens AB, Coon D, Wisniewski S, *et al*. Measurement of leisure time satisfaction in family caregivers. *Aging Ment Health* 2004;**8**:450-9.

For peer review only

29. Smith TO, Hawker GA, Hunter DJ, *et al*. The OMERACT-OARSI Core Domain Set for Measurement in Clinical Trials of Hip and/or Knee Osteoarthritis. *J Rheumatol* 2019;**46**:981-9.

30. Chiarotto A, Deyo RA, Terwee CB, *et al*. Core outcome domains for clinical trials in non-specific low back pain. *Eur Spine J* 2015;**24**:1127-42.

31. Klokkerud M, Dagfinrud H, Uhlig T, et al. Developing and testing a consensus-based core set of outcome measures for rehabilitation in musculoskeletal diseases. *Scand J Rheumatol* 2018;**47**:225-34.

32. Teare MD, Dimairo M, Shephard N, Hayman A, Whitehead A, Walters SJ. Sample size requirements to estimate key design parameters from external pilot randomised controlled trials: a simulation study. *Trials* 2014;**15**:264.

33. Whitehead AL, Julious SA, Cooper CL, Campbell MJ. Estimating the sample size for a pilot randomised trial to minimise the overall trial sample size for the external pilot and main trial for a continuous outcome variable. *Stat Methods Med Res* 2016;**25**:1057-73.

34. Sim J, Lewis M. The size of a pilot study for a clinical trial should be calculated in relation to considerations of precision and efficiency. *J Clin Epidemiol* 2012;**65**:301–8.

35. Avery KN, Williamson PR, Gamble C, *et al*. Informing efficient randomised controlled trials: exploration of challenges in developing progression criteria for internal pilot studies. *BMJ Open* 2017;**7**:e013537.

36. Malterud K, Siersma VD, Guassora AD. Sample size in qualitative interview studies: guided by information power. *Qual Health Res* 2016;**26**:1753-60.

37. Sekhon M, Cartwright M, Francis JJ. Acceptability of healthcare interventions: an overview of reviews and development of a theoretical framework. *BMC Health Serv Res* 2017;**17**:88.

38. Craig P, Dieppe P, Macintyre S, *et al*. Developing and evaluating complex interventions: the new Medical Research Council guidance. *BMJ* 2008;**337**:a1655.

39. Moore GF, Audrey S, Barker M, *et al*. Process evaluation of complex interventions: Medical Research Council guidance. *BMJ* 2015;**350**:h1258.

40. Skivington K, Matthews L, Simpson SA, *et al*. A new framework for developing and evaluating complex interventions: update of Medical Research Council guidance. *BMJ* 2021;374:n2061.

41. Data Protection Act. 2018. Data accessed: 06Dec2022. Available at: http://www.legislation.gov.uk/ukpga/2018/12/contents/enacted.

42. Schulz KF, Altman DG, Moher D; CONSORT Group. CONSORT 2010 statement: updated guidelines for reporting parallel group randomised trials. *PLoS Med* 2010;**7**:e1000251.

43. Hoffmann TC, Glasziou PP, Boutron I, et al. Better reporting of interventions: template for intervention description and replication (TIDieR) checklist and guide. *BMJ* 2014;**348**:g1687.

For peer review only

For peer review only

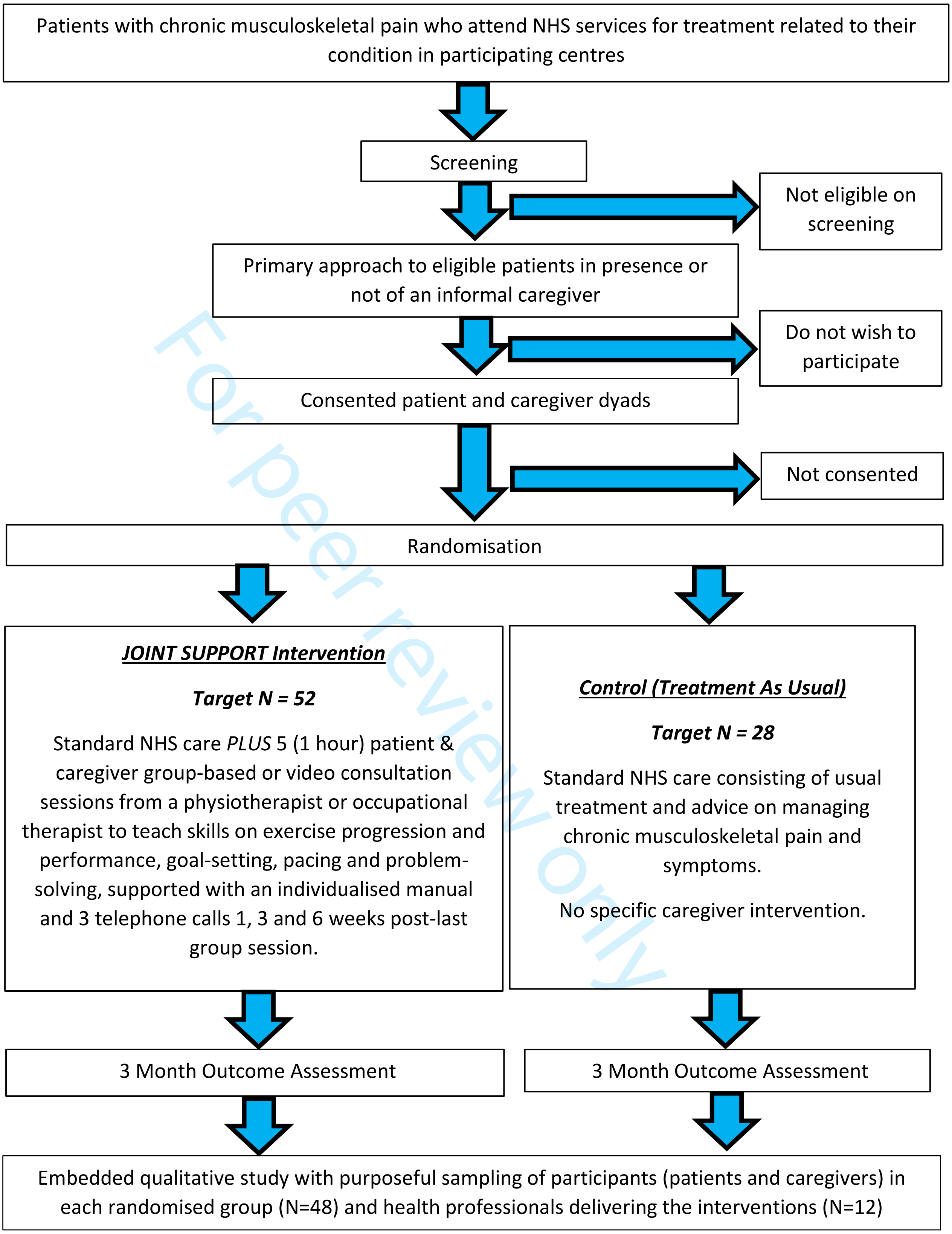

For peer review only

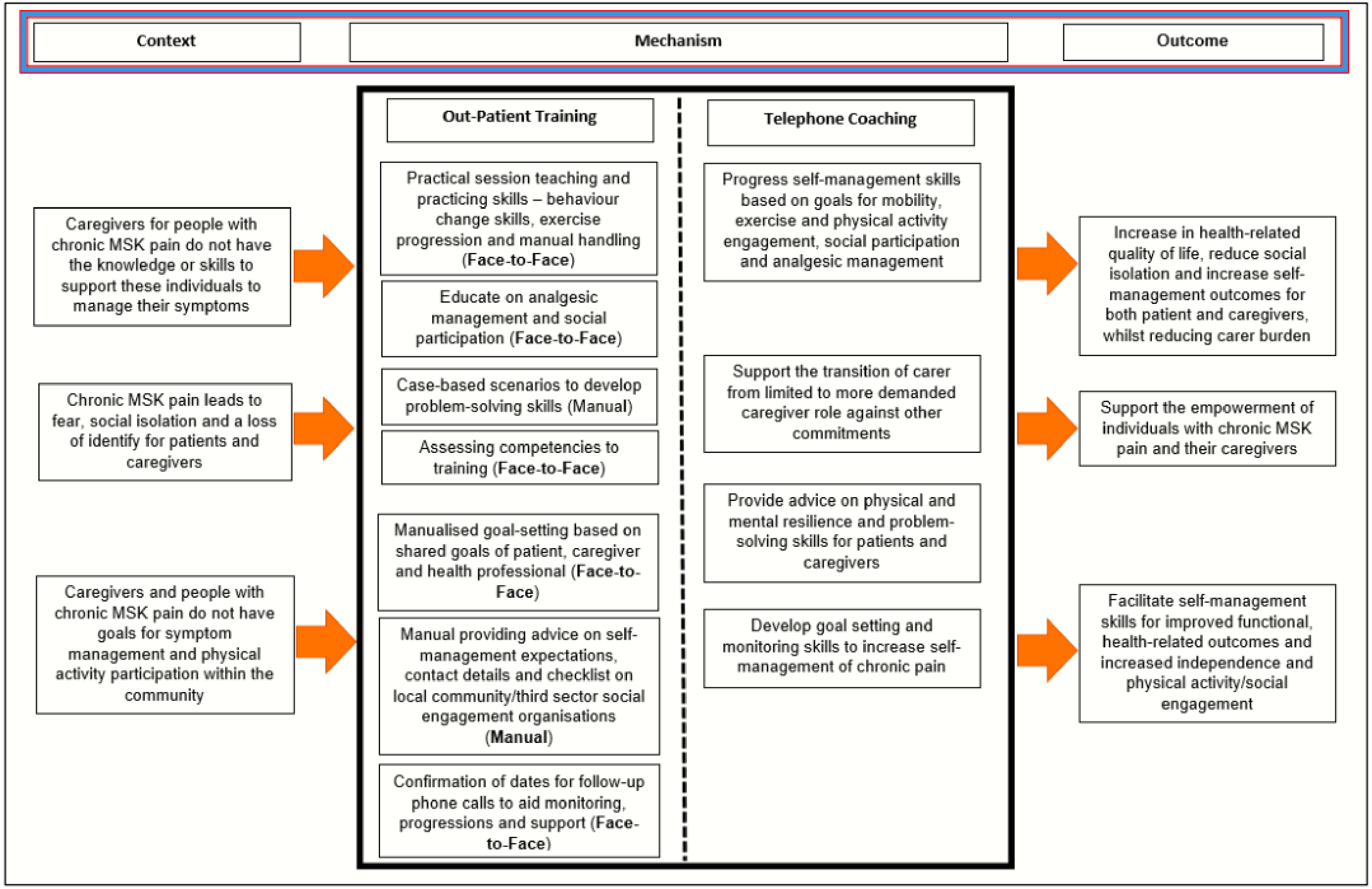
Context
Mechanism
Outcome
Out-Patient Training
Telephone Coaching
Caregivers for people with chronic MSK pain do not have the knowledge or skills to support these individuals to manage their symptoms
Chronic MSK pain leads to fear, social isolation and a loss of identify for patients and caregivers
Caregivers and people with chronic MSK pain do not have goals for symptom management and physical activity participation within the community
Practical session teaching and practicing skills – behaviour change skills, exercise progression and manual handling (Face-to-Face)
Educate on analgesic management and social participation (Face-to-Face)
Case-based scenarios to develop problem-solving skills (Manual)
Assessing competencies to training (Face-to-Face)
Manualised goal-setting based on shared goals of patient, caregiver and health professional (Face-to-Face)
Manual providing advice on self-management expectations, contact details and checklist on local community/third sector social engagement organisations (Manual)
Confirmation of dates for follow-up phone calls to aid monitoring, progressions and support (Face-to-Face)
Progress self-management skills based on goals for mobility, exercise and physical activity engagement, social participation and analgesic management
Support the transition of carer from limited to more demanded caregiver role against other commitments
Provide advice on physical and mental resilience and problem-solving skills for patients and caregivers
Develop goal setting and monitoring skills to increase self-management of chronic pain
Increase in health-related quality of life, reduce social isolation and increase self-management outcomes for both patient and caregivers, whilst reducing carer burden
Support the empowerment of individuals with chronic MSK pain and their caregivers
Facilitate self-management skills for improved functional, health-related outcomes and increased independence and physical activity/social engagement

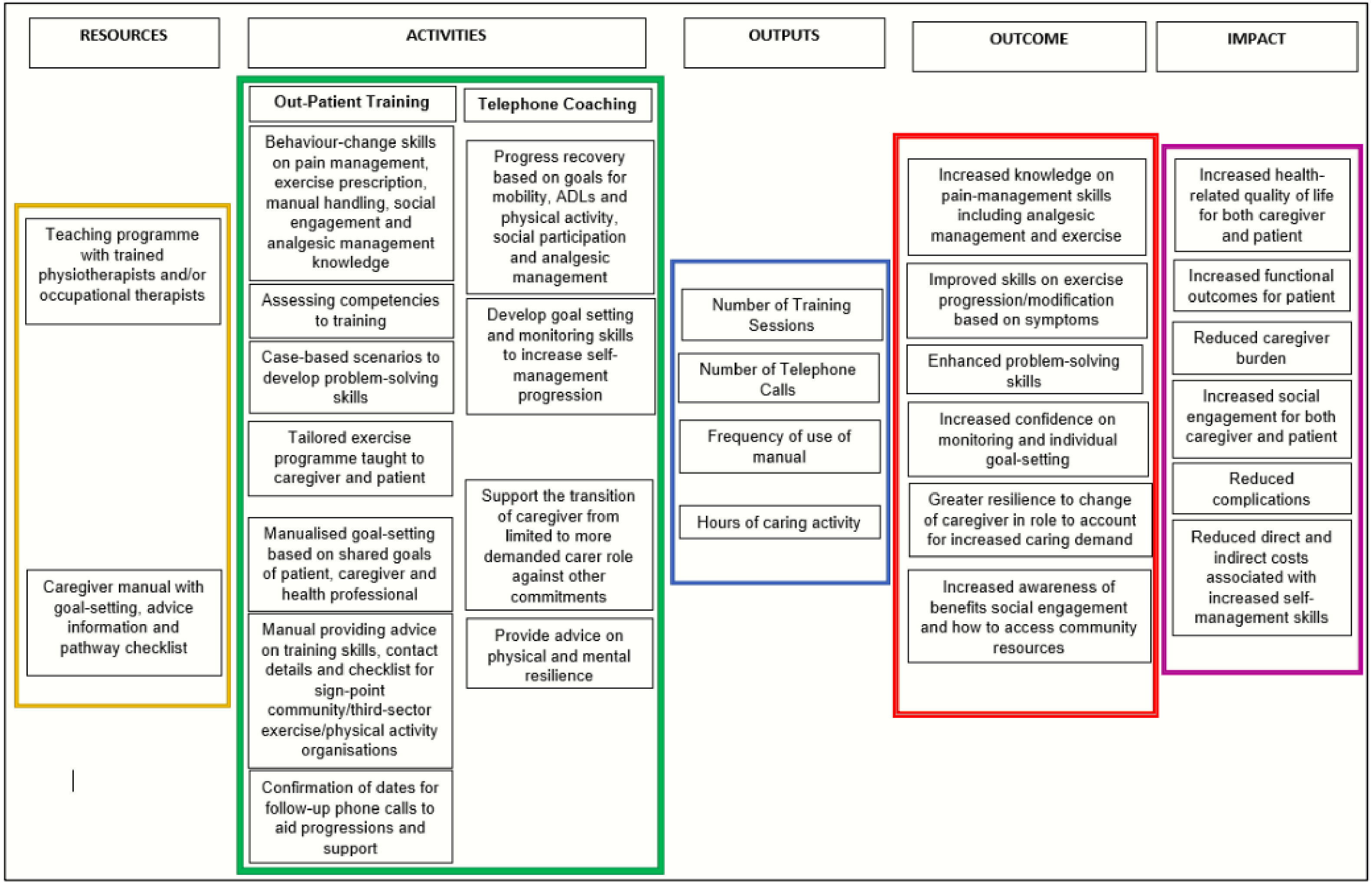
RESOURCES
ACTIVITIES
OUTPUTS
OUTCOME
IMPACT
Teaching programme with trained physiotherapists and/or occupational therapists
Caregiver manual with goal-setting, advice information and pathway checklist
Out-Patient Training
Behaviour-change skills on pain management, exercise prescription, manual handling, social engagement and analgesic management knowledge
Assessing competencies to training
Case-based scenarios to develop problem-solving skills
Tailored exercise programme taught to caregiver and patient
Manualised goal-setting based on shared goals of patient, caregiver and health professional
Manual providing advice on training skills, contact details and checklist for sign-point community/third-sector exercise/physical activity organisations
Confirmation of dates for follow-up phone calls to aid progressions and support
Telephone Coaching
Progress recovery based on goals for mobility, ADLs and physical activity, social participation and analgesic management
Develop goal setting and monitoring skills to increase self-management progression
Support the transition of caregiver from limited to more demanded carer role against other commitments
Provide advice on physical and mental resilience
Number of Training Sessions
Number of Telephone Calls
Frequency of use of manual
Hours of caring activity
Increased knowledge on pain-management skills including analgesic management and exercise
Improved skills on exercise progression/modification based on symptoms
Enhanced problem-solving skills
Increased confidence on monitoring and individual goal-setting
Greater resilience to change of caregiver in role to account for increased caring demand
Increased awareness of benefits social engagement and how to access community resources
Increased health-related quality of life for both caregiver and patient
Increased functional outcomes for patient
Reduced caregiver burden
Increased social engagement for both caregiver and patient
Reduced complications
Reduced direct and indirect costs associated with increased self-management skills

SPIRIT 2013 Checklist: Recommended items to address in a clinical trial protocol and related documents*

Section/item	Item No	Description	In Paper
**Administrative information**			
Title	1	Descriptive title identifying the study design, population, interventions, and, if applicable, trial acronym	Title; Title Page, Pg 1, Line 1
Trial registration	2a	Trial identifier and registry name. If not yet registered, name of intended registry	Trial Registration Number; Abstract, Pg 2, Para 4
	2b	All items from the World Health Organization Trial Registration Data Set	Title Page; Pg 2, Para 4/ Acknowledgements & Declarations, Pg 16, Para 9
Protocol version	3	Date and version identifier	Acknowledgements & Declarations Pg 16, Para 6

For peer review only

Funding	4	Sources and types of financial, material, and other support	Acknowledgements & Declarations Pg 16, Para 4
Roles and responsibilities	5a	Names, affiliations, and roles of protocol contributors	Acknowledgements & Declarations Pg 16, Para 7
	5b	Name and contact information for the trial sponsor	Acknowledgements & Declarations Pg 16, Para 3
	5c	Role of study sponsor and funders, if any, in study design; collection, management, analysis, and interpretation of data; writing of the report; and the decision to submit the report for publication, including whether they will have ultimate authority over any of these activities	Acknowledgements & Declarations Pg 16, Para 4-5
	5d	Composition, roles, and responsibilities of the coordinating centre, steering committee, endpoint adjudication committee, data management team, and other individuals or groups overseeing the trial, if applicable (see Item 21a for data monitoring committee)	Acknowledgements & Declarations Pg 16, Para 2-3
**Introduction**			

For peer review only

Background and rationale	6a	Description of research question and justification for undertaking the trial, including summary of relevant studies (published and unpublished) examining benefits and harms for each intervention	Introduction, Pg 3, Para 1 to 5
	6b	Explanation for choice of comparators	Methods & Analysis, Intervention, Usual Care Pg 6, Para 1-2
Objectives	7	Specific objectives or hypotheses	Aims & Objectives; Objectives, Pg 3 & 4, Para 1-2
Trial design	8	Description of trial design including type of trial (eg, parallel group, crossover, factorial, single group), allocation ratio, and framework (eg, superiority, equivalence, noninferiority, exploratory)	Methods & Analysis, Trial Design, Pg 4, Para 1-2
**Methods: Participants, interventions, and outcomes**			
Study setting	9	Description of study settings (eg, community clinic, academic hospital) and list of countries where data will be collected. Reference to where list of study sites can be obtained	Methods & Analysis, Recruitment; Pg 5, Para 1 / Acknowledgements & Declarations Pg 16, Para 1

Eligibility criteria	10	Inclusion and exclusion criteria for participants. If applicable, eligibility criteria for study centres and individuals who will perform the interventions (eg, surgeons, psychotherapists)	Methods & Analysis; Eligibility Pg 4 & 5 Bullets 1-5
Interventions	11a	Interventions for each group with sufficient detail to allow replication, including how and when they will be administered	Methods & Analysis; Intervention, Pg 6, 7 & 8, Para 1-10
	11b	Criteria for discontinuing or modifying allocated interventions for a given trial participant (eg, drug dose change in response to harms, participant request, or improving/worsening disease)	Ethics and Dissemination, Pg 15, Para 1
	11c	Strategies to improve adherence to intervention protocols, and any procedures for monitoring adherence (eg, drug tablet return, laboratory tests)	Methods & Analysis; Quality Assessment, Pg 9, Para 1 & 2
	11d	Relevant concomitant care and interventions that are permitted or prohibited during the trial	Methods & Analysis; Intervention; Co-interventions, Pg 8, Para 1

Outcomes	12	Primary, secondary, and other outcomes, including the specific measurement variable (eg, systolic blood pressure), analysis metric (eg, change from baseline, final value, time to event), method of aggregation (eg, median, proportion), and time point for each outcome. Explanation of the clinical relevance of chosen efficacy and harm outcomes is strongly recommended	Methods & Analysis, Assessments; Pg 9 & 10, Bullets 1-8
Participant timeline	13	Time schedule of enrolment, interventions (including any run-ins and washouts), assessments, and visits for participants. A schematic diagram is highly recommended (see Figure)	Figure 4
Sample size	14	Estimated number of participants needed to achieve study objectives and how it was determined, including clinical and statistical assumptions supporting any sample size calculations	Data Analysis, Sample Size, Pg 10, Para 1
Recruitment	15	Strategies for achieving adequate participant enrolment to reach target sample size	Data Analysis, Sample Size Pg 10, Para 1
**Methods: Assignment of interventions (for controlled trials)**			
Allocation:			
Sequence generation	16a	Method of generating the allocation sequence (eg, computer-generated random numbers), and list of any factors for stratification. To reduce predictability of a random sequence, details of any planned restriction (eg, blocking) should be provided in a separate document that is unavailable to those who enrol participants or assign interventions	Randomisation , blinding and allocation concealment, Pg 5 & 6, Para 1 & 2

Allocation concealment mechanism	16b	Mechanism of implementing the allocation sequence (eg, central telephone; sequentially numbered, opaque, sealed envelopes), describing any steps to conceal the sequence until interventions are assigned	Randomisation, blinding and allocation concealment, Pg 5 & 6, Para 1 & 2
Implementation	16c	Who will generate the allocation sequence, who will enrol participants, and who will assign participants to interventions	Randomisation, blinding and allocation concealment, Pg 5 & 6, Para 1 & 2
Blinding (masking)	17a	Who will be blinded after assignment to interventions (eg, trial participants, care providers, outcome assessors, data analysts), and how	Randomisation, blinding and allocation concealment Pg 6, Para 3
	17b	If blinded, circumstances under which unblinding is permissible, and procedure for revealing a participant's allocated intervention during the trial	Not applicable
**Methods: Data collection, management, and analysis**			
Data collection methods	18a	Plans for assessment and collection of outcome, baseline, and other trial data, including any related processes to promote data quality (eg, duplicate measurements, training of assessors) and a description of study instruments (eg, questionnaires, laboratory tests) along with their reliability and validity, if known. Reference to where data collection forms can be found, if not in the protocol	Methods & Analysis, Assessments Pg 11, Para 1

	18b	Plans to promote participant retention and complete follow-up, including list of any outcome data to be collected for participants who discontinue or deviate from intervention protocols	Methods & Analysis, Assessments Pg 11, Para 1
Data management	19	Plans for data entry, coding, security, and storage, including any related processes to promote data quality (eg, double data entry; range checks for data values). Reference to where details of data management procedures can be found, if not in the protocol	Methods & Analysis, Assessments; Pg 11, Para 1 Quality Assessments Pg 9, Para 1
Statistical methods	20a	Statistical methods for analysing primary and secondary outcomes. Reference to where other details of the statistical analysis plan can be found, if not in the protocol	Data Analysis Pg 11, Para 1
	20b	Methods for any additional analyses (eg, subgroup and adjusted analyses)	Data Analysis Pg 11, Para 1
	20c	Definition of analysis population relating to protocol non-adherence (eg, as randomised analysis), and any statistical methods to handle missing data (eg, multiple imputation)	Not applicable
**Methods: Monitoring**			
Data monitoring	21a	Composition of data monitoring committee (DMC); summary of its role and reporting structure; statement of whether it is independent from the sponsor and competing interests; and reference to where further details about its charter can be found, if not in the protocol. Alternatively, an explanation of why a DMC is not needed	Acknowledgements & Declarations Pg 16, Para 2

	21b	Description of any interim analyses and stopping guidelines, including who will have access to these interim results and make the final decision to terminate the trial	None.
Harms	22	Plans for collecting, assessing, reporting, and managing solicited and spontaneously reported adverse events and other unintended effects of trial interventions or trial conduct	Ethics and Dissemination, Pg 15, Para 1
Auditing	23	Frequency and procedures for auditing trial conduct, if any, and whether the process will be independent from investigators and the sponsor	Quality Assessment Pg 9, Para 1
**Ethics and dissemination**			
Research ethics approval	24	Plans for seeking research ethics committee/institutional review board (REC/IRB) approval	Acknowledgem ents & Declarations Pg 16, Para
Protocol amendments	25	Plans for communicating important protocol modifications (eg, changes to eligibility criteria, outcomes, analyses) to relevant parties (eg, investigators, REC/IRBs, trial participants, trial registries, journals, regulators)	Ethics and Dissemination Pg 15, Para 1 & 2
Consent or assent	26a	Who will obtain informed consent or assent from potential trial participants or authorised surrogates, and how (see Item 32)	Methods & Analysis, Recruitment Pg 5, Para 1
	26b	Additional consent provisions for collection and use of participant data and biological specimens in ancillary studies, if applicable	Not applicable

For peer review only

Confidentiality	27	How personal information about potential and enrolled participants will be collected, shared, and maintained in order to protect confidentiality before, during, and after the trial	Ethics and Dissemination Pg 15, Para 1
Declaration of interests	28	Financial and other competing interests for principal investigators for the overall trial and each study site	Acknowledgements & Declarations, Pg 16, Para 9
Access to data	29	Statement of who will have access to the final trial dataset, and disclosure of contractual agreements that limit such access for investigators	Acknowledgements & Declarations; Pg 16, Para 11; Consent Form
Ancillary and post-trial care	30	Provisions, if any, for ancillary and post-trial care, and for compensation to those who suffer harm from trial participation	Not applicable
Dissemination policy	31a	Plans for investigators and sponsor to communicate trial results to participants, healthcare professionals, the public, and other relevant groups (eg, via publication, reporting in results databases, or other data sharing arrangements), including any publication restrictions	Ethics and Dissemination Pg 15, Para 2
	31b	Authorship eligibility guidelines and any intended use of professional writers	Not applicable
	31c	Plans, if any, for granting public access to the full protocol, participant-level dataset, and statistical code	Acknowledgements & Declarations, Pg 16, Para 11

For peer review only

**Appendices**			
Informed consent materials	32	Model consent form and other related documentation given to participants and authorised surrogates	Uploaded with submission
Biological specimens	33	Plans for collection, laboratory evaluation, and storage of biological specimens for genetic or molecular analysis in the current trial and for future use in ancillary studies, if applicable	Not applicable

*It is strongly recommended that this checklist be read in conjunction with the SPIRIT 2013 Explanation & Elaboration for important clarification on the items. Amendments to the protocol should be tracked and dated. The SPIRIT checklist is copyrighted by the SPIRIT Group under the Creative Commons "Attribution-NonCommercial-NoDerivs 3.0 Unported" license.

For peer review only
