# Supplementary material for: Informal caregiver training for people with chronic pain in musculoskeletal services (JOINT SUPPORT): protocol for a feasibility randomised controlled trial
Source: BMJ Open. 2023 Jan 27;13(1):e070865. doi: 10.1136/bmjopen-2022-070865 (PMC9884939; doi:10.1136/bmjopen-2022-070865)
Supplement: Reviewer comments [file bmjopen-2022-070865.reviewer_comments.pdf]

PEER REVIEW HISTORY

BMJ Open publishes all reviews undertaken for accepted manuscripts. Reviewers are asked to complete a checklist review form (**http://bmjopen.bmj.com/site/about/resources/checklist.pdf**) and are provided with free text boxes to elaborate on their assessment. These free text comments are reproduced below.

ARTICLE DETAILS

**TITLE (PROVISIONAL)**	Informal caregiver training for people with chronic pain in musculoskeletal services (JOINT SUPPORT): protocol for a feasibility randomised controlled trial
**AUTHORS**	Smith, Toby; Khoury, Reema; Ashford, Polly-Anna; Hanson, Sarah; Welsh, Allie; Clark, Allan; Dures, Emma; Adams, Jo

This article was not externally reviewed at BMJ Open. Protocols that have been independently assessed prior to submission to BMJ Open are usually fast-tracked to publication on the grounds that further substantial changes will not be possible. This independent assessment will usually be external, independent review for both a substantial grant award from a non-commercial or government funder and ethics approval.
